# Supplementary material for: Midwives’ survey of their weight management practice before and after the GLOWING guideline implementation intervention: A pilot cluster randomised controlled trial
Source: PLoS One. 2023 Jan 20;18(1):e0280624. doi: 10.1371/journal.pone.0280624 (PMC9858407; doi:10.1371/journal.pone.0280624)
Supplement: S1 File — (DOCX) [file pone.0280624.s001.docx]

GLOWING Pilot Cluster RCT Protocol

Full Title: GestationaL Obesity Weight management: Implementation of National Guidelines (GLOWING): A pilot cluster randomised controlled trial of a guideline implementation intervention for the management of maternal obesity by midwives

Short Title: GLOWING Pilot Cluster RCT

Trial registration: To be registered on the National Institute for Health

Research (NIHR) Portfolio database

Funders: NIHR Postdoctoral Research Fellowship

PDF-2011-04-034

Sponsor: Newcastle upon Tyne Hospitals NHS Foundation Trust

# Protocol contacts

***Chief Investigator:***

Dr Nicola Heslehurst (Lecturer, NIHR Postdoctoral Research Fellow)

Institute of Health & Society

Newcastle University

Baddiley-Clark Building

Richardson Road

Newcastle upon Tyne, NE2 4AX

Tel: +44 (0) 191 208 3823, Email: [nicola.heslehurst@ncl.ac.uk](mailto:nicola.heslehurst@ncl.ac.uk)

***Co-Investigators:***

Prof Elaine McColl Prof Judith Rankin

Director Newcastle Clinical Trials Unit Prof Maternal and Perinatal Epidemiology

Institute of Health & Society Institute of Health & Society

Newcastle Clinical Trials Unit Newcastle University

1-4 Claremont Terrace Baddiley-Clark Building, Richardson Road

Newcastle upon Tyne, NE2 4AE Newcastle upon Tyne, NE2 4AX

+44 (0) 191 208 7260 +44 (0) 191 208 5267

[elaine.mccoll@ncl.ac.uk](mailto:elaine.mccoll@ncl.ac.uk) [judith.rankin@ncl.ac.uk](mailto:judith.rankin@ncl.ac.uk)

Dr Catherine McParlin

Project Research Midwife/Midwife Facilitator for the Intervention

Institute of Health & Society/Newcastle University Teaching Hospitals NHS Trust

Newcastle University

Baddiley-Clark Building

Richardson Road

Newcastle upon Tyne, NE2 4AX

Tel: c/o +44 (0) 191 208 3823, Email: [Catherine.McParlin@ncl.ac.uk](mailto:Catherine.McParlin@ncl.ac.uk)

***Health Psychology:***

Prof Falko Sniehotta

Institute of Health & Society

Newcastle University

Baddiley-Clark Building

Richardson Road

Newcastle upon Tyne, NE2 4AX

Tel: +44 (0) 191 208 3815, Email: [falko.sniehotta@ncl.ac.uk](mailto:falko.sniehotta@ncl.ac.uk)

***Health Economics:***

Prof Luke Vale Supported by: Dr Stephen Rice

Institute of Health & Society Institute of Health & Society

Newcastle University Newcastle University

Baddiley-Clark Building Baddiley-Clark Building

Richardson Road Richardson Road

Newcastle upon Tyne, NE2 4AX Newcastle upon Tyne, NE2 4AX

+44 0191 2085590 +44 (0) 191 208 3800

[luke.vale@ncl.ac.uk](mailto:luke.vale@ncl.ac.uk) [Stephen.Rice@ncl.ac.uk](mailto:Stephen.Rice@ncl.ac.uk)

***Statistics:***

Denise Howel

Senior Lecturer in Epidemiological Statistics

Institute of Health & Society

Newcastle University

Baddiley-Clark Building

Richardson Road

Newcastle upon Tyne, NE2 4AX

Tel: 0191 208 7381/7245, Email: [denise.howel@ncl.ac.uk](mailto:denise.howel@ncl.ac.uk)

***Sponsor:***

Newcastle upon Tyne Hospitals NHS Foundation Trust

Newcastle Joint Research Office (NJRO)

Regent Point

Regent Farm Road

Gosforth

Newcastle upon Tyne NE3 3HD

Contact: Ms Amanda Tortice

Research Operations Manager

Tel: 0191 282 5959, Email: [amanda.tortice@ncl.ac.uk](mailto:amanda.tortice@ncl.ac.uk)

***Funders:***

National Institute for Health Research (NIHR) Postdoctoral Fellowship

NIHR Trainees Coordinating Centre

Leeds Innovation Centre

103 Clarendon Road

Leeds LS2 9DF

Tel: +44 (0) 113 346 6260, Email: [nihrfellowshipenquiries@nihrtcc.org.uk](mailto:nihrfellowshipenquiries@nihrtcc.org.uk)

***External Project Advisory Group:***

1. Prof Marian Knight

National Perinatal Epidemiology Unit

Nuffield Department of Population Health

University of Oxford

Oxford, OX3 7LF

Tel: +44 (0)1865 289700, Email: [marian.knight@npeu.ox.ac.uk](mailto:marian.knight@npeu.ox.ac.uk)

1. Prof Janet Shucksmith

Prof of Public Health/ Assistant Dean (Research)

Health & Social Care Institute

School of Health & Social Care

Teesside University

Middlesbrough, TS1 3BA

Tel: +44 (0) 1642 342969, Email: [j.shucksmith@tees.ac.uk](mailto:j.shucksmith@tees.ac.uk)

1. Prof Carolyn Summerbell

Prof of Human Nutrition/ Principal of John Snow College

School of Medicine, Pharmacy and Health

Durham University

Queen's Campus

Stockton on Tees, TS17 6BH

Tel: +44 (0) 191 33 40071, Email: [carolyn.summerbell@durham.ac.uk](mailto:carolyn.summerbell@durham.ac.uk)

1. Tommy’s the Baby Charity

Represented by Dr Beckie Lang

Health and Research Manager

Nicholas House

3 Laurence Pountney Hill

London, EC4R 0BB

Tel: 07887 617195, Email: [BLang@tommys.org](mailto:BLang@tommys.org)

1. Royal College of Midwives

Represented by Janet Fyle Professional Policy Advisor

15 Mansfield St

London, W1G 9NH

Tel: +44 (0) 20 7312 3465, Email: [Janet.Fyle@rcm.org.uk](mailto:Janet.Fyle@rcm.org.uk)

1. National Institute for Health and Care Excellence

Represented by Julie Royce

Associate Director Implementation Support

National Institute for Health and Care Excellence

10 Spring Gardens

London, SW1A 2BU

Tel: +44 (0) 300 323 0140, Email: [Julie.Royce@nice.org.uk](mailto:Julie.Royce@nice.org.uk)

Represented by Alexa Forrester

Implementation Adviser

National Institute for Health and Care Excellence

Level 1A City Tower

Piccadilly Plaza

Manchester, M1 4BD

Tel: +44 (0)161 870 3270, Email: [Alexa.Forrester@nice.org.uk](mailto:Alexa.Forrester@nice.org.uk)

1. Prof Lucilla Poston (PI on NIHR funded UPBEAT intervention for maternal obesity)

Prof of Maternal & Fetal Health

Kings College London

10 W 4.04, 10th Floor North Wing

St Thomas's Hospital

Westminster Bridge Road

London, SE1 7EH

Tel: +44 (0) 20 7188 3644, Email: [lucilla.poston@kcl.ac.uk](mailto:lucilla.poston@kcl.ac.uk)

1. Dr Diane Farrer

Research Midwife, NIHR Postdoctoral Research Fellow

Bradford Institute for Health Research

Bradford Royal Infirmary

Duckworth Lane

Bradford, BD9 6RJ

Tel: +44 (0) 1274 38 3416, Email: [Diane.Farrar@bthft.nhs.uk](mailto:Diane.Farrar@bthft.nhs.uk)

# Protocol signature

***Signature Date 05/05/2017***

***Dr Nicola Heslehurst, Chief Investigator***

# Contents

[1. Protocol contacts 1](#_Toc481751176)

[2. Protocol signature 3](#_Toc481751177)

[3. Contents 4](#_Toc481751178)

[4. Glossary of Abbreviations 5](#_Toc481751179)

[5. Responsibilities 6](#_Toc481751180)

[6. Protocol Summary 7](#_Toc481751181)

[7. Background 8](#_Toc481751182)

[8. Objectives 11](#_Toc481751183)

[9. Study Design 12](#_Toc481751184)

[10. Participants 15](#_Toc481751185)

[11. Screening, Recruitment and Consent 16](#_Toc481751186)

[12. Study Intervention Details 18](#_Toc481751187)

[13. Randomisation 20](#_Toc481751188)

[14. Blinding 20](#_Toc481751189)

[15. Study Data 20](#_Toc481751190)

[16. Statistical & Analytical Considerations 24](#_Toc481751191)

[17. Compliance and Withdrawal 24](#_Toc481751192)

[18. Data Monitoring, Quality Control and Quality Assurance 25](#_Toc481751193)

[19. Adverse Event Monitoring and Reporting 26](#_Toc481751194)

[20. Ethics & Regulatory Issues 26](#_Toc481751195)

[21. Confidentiality 26](#_Toc481751196)

[22. Insurance and Finance 26](#_Toc481751197)

[23. Study Report / Publications 26](#_Toc481751198)

[24. References 28](#_Toc481751199)

[25. Appendices 31](#_Toc481751200)

# Glossary of Abbreviations

| ***Abbreviation*** | ***Definition*** |
| --- | --- |
| *BMI* | *Body mass index* |
| *CI* | *Chief Investigator* |
| *CSP* | *Centralised System for Permissions* |
| *EPOC* | *Effective Practice and Organisation of Care* |
| *FFQ* | *Food Frequency Questionnaire* |
| *GCP* | *Good Clinical Practice* |
| *GLOWING* | *GestationaL Obesity Weight management: Implementation of National Guidelines* |
| *ICC* | *Intra Cluster Correlation* |
| *MRC* | *Medical Research Council* |
| *NHS* | *National Health Service* |
| *NICE* | *National Institute for Health and Care Excellence* |
| *NIHR* | *National Institute for Health Research* |
| *NUTH NHS Trust* | *Newcastle upon Tyne Hospitals NHS Foundation Trust* |
| *RCM* | *Royal College of Midwives* |
| *PAG* | *Project Advisory Group* |
| *PI* | *Principal Investigator* |
| *PIS* | *Participant Information Sheet* |
| *PPAQ* | *Pregnancy Physical Activity Questionnaire* |
| *R&D* | *Research & Development* |
| *RCT* | *Randomised Controlled Trial* |
| *SCT* | *Social Cognitive Theory* |
| *TACT* | *Target, Action, Context, Time* |

# Responsibilities

**Sponsor:** NUTH NHS Foundation Trust will act as the sponsor for this study.

**Funder:** NIHR are funding this study.

**Trial Management:** An external Project Advisory Group (PAG) has been established to advise on the development of the intervention, outcome measures, and to oversee the progress of the pilot study.

The day-to-day management of the trial will be co-ordinated by Dr Nicola Heslehurst

***Principal Investigator:*** *The local Principal Investigator at each site will have overall responsibility for the conduct of the study at a particular trial site*

***Trial Management:***

*The following functions falling under the responsibility of the sponsor will be delegated to Dr Nicola Heslehurst [Chief Investigator]:*

- *Ethics Committee Opinion (including application for research ethics committee favourable opinion, notification of protocol amendments and end of trial, site specific assessment & local approval)*
- *R&D Approval (including application for global checks, via NIHR CSP)*
- *Good Clinical Practice and Trial Conduct (including GCP arrangements, data monitoring, emergency & safety procedures)*
- *Administration of funding for the study*

***Trial conduct at site:***

***Investigator responsibilities:***

- *Study conduct and the welfare of study subjects*
- *Familiarity with the study intervention(s)*
- *Compliance with the protocol, documentation of any protocol deviations and reporting of all serious adverse events*
- *Screening and recruitment of subjects*
- *Obtaining local approval and abiding by the policies of Research Governance*
- *Compliance with the Principles of GCP, the Research Governance Framework for Health and Social Care, the Data Protection Act and any other relevant legislation and regulatory guidance.*
- *Ensuring that no participant is recruited into the study until all relevant regulatory permissions and approvals have been obtained.*
- *Obtaining written informed consent from participants prior to any study specific procedures.*
- *The Principal Investigator (PI) shall be qualified by education, training and experience to assume responsibility for the proper conduct of the trial. S/he shall provide a current signed & dated curriculum vitae as evidence for the Trial Master File.*
- *Ensuring Study Site team members are appropriately qualified by education, training and experience to undertake the conduct of the study.*
- *Availability for Investigator meetings, monitoring visits and in the case of an audit.*
- *Maintaining study documentation and compliance with reporting requests*
- *Maintaining a site file, including copies of study approval, list of subjects and their signed informed consent forms*
- *Documenting appropriate delegation of tasks to other study personnel e.g. Research Nurse, Co-Investigator(s), Trial Coordinators, Data Managers*
- *Ensuring data collected is accurate, timely & complete*
- *Providing updates on the progress of the trial*
- *Ensuring subject confidentiality is maintained during the project and archival period*
- *Ensuring archival of study documentation for 10 years following the end of the study*

# Protocol Summary

***Short title:*** *GLOWING Pilot Cluster RCT*

***Protocol version:*** *4.0*

***Protocol date:*** *05/05/2017*

***Chief investigator:*** *Dr Nicola Heslehurst*

***Sponsor:*** *NUTH NHS Trust*

***Funder:*** *NIHR*

***Study design:*** *Pilot Cluster RCT*

***Study intervention:*** *Intensive one day training for midwives on weight communication and weight management in pregnancy*

***Primary objective (intervention):*** *Implementation of guidelines into clinical practice through midwives behaviour change*

***Secondary objective (intervention):*** *Identify if midwives’ practice mediates pregnant/postnatal women’s weight, or weight-related behaviours*

***Primary outcome (intervention):*** *Midwives’ behaviours in practice*

***Pilot RCT objectives:*** *1) Pilot the intervention delivery, data collection and analysis methods to ascertain feasibility and acceptability thereof*

*2) Identify the intervention’s active ingredients (in success or failure) through process evaluation*

*3) Collect data required to inform sample size estimations and scope data collection procedures for economic evaluation within the definitive trial*

***Number of study sites:*** *Four*

***Study population/size:*** Intervention delivery (training) population:

- *All community midwives in the intervention arm (2 sites)*
- *Any hospital-based midwives with a specific obesity or weight management role*

Outcome measures population:

*1) Community midwives/18 per site*

*2) Pregnant/postnatal women/30 per site*

***Study duration:*** *Two years*

# Background

***7.1 Rational and scientific justification for the research:***

First trimester maternal obesity (body mass index (BMI)>30kg/m^2^) in England has doubled from 7.6% in 1989 (approximately 45,000 women) to 15.6% in 2007 (approximately 92,500 women), and is significantly associated with inequalities including area of residence deprivation, ethnic minority groups, and unemployment [[1](#_ENREF_1) [2](#_ENREF_2)]. Maternal obesity has short and long term impacts for women and babies, including maternal and neonatal mortality, gestational diabetes, thromboembolism, infection, haemorrhage, reduced breast feeding, congenital anomalies, and obesity development in offspring [[3-8](#_ENREF_3)]. However, pregnancy is often cited as a life-course opportunity for intervention to address personal behaviour change and obesity: it is a period of metabolic plasticity, significant shift in attitude and spontaneous change in behaviour; women are more receptive to nutrition advice during pregnancy; and it addresses obesity for the next generation earlier than do childhood obesity strategies [[9](#_ENREF_9) [10](#_ENREF_10)]. NICE also identifies pregnancy as a key time for health professionals to advise and support women as it is a vulnerable life stage for increased risk of weight gain, and a key life stage when people are more open to change [[11](#_ENREF_11) [12](#_ENREF_12)].

Management of maternal obesity is increasingly included in public health and clinical guidelines in the UK and internationally[[13](#_ENREF_13) [14](#_ENREF_14)], and midwives are expected to have a public health role which includes addressing obesity. NICE evidence-based guidelines for weight management before, during and after pregnancy include recommendations for health professional provision of advice and support to obese women including discussing obesity risks, weight related behaviour, incorporating practical and tailored advice, and being sensitive to women’s weight concerns [[15](#_ENREF_15)].

The pressures of managing an increasing burden of maternal obesity are not purely resource based, although there are significant resource implications [[5](#_ENREF_5) [16-18](#_ENREF_16)]. Health professionals lack confidence in their own expertise to provide advice and support for obese women during pregnancy, and face difficulties in discussing obesity due to its sensitive nature[[19](#_ENREF_19)]. This adds to concerns about communicating risk to women who may already feel disempowered, resulting in inequalities in the level of support for obese pregnant women due to inconsistent and ad hoc advice [[1](#_ENREF_1) [20-22](#_ENREF_20)]. Obese patients have described health professionals’ communication as ambivalent, insulting, judgemental, insensitive, and patronising [[23-26](#_ENREF_23)]. Negative experiences have led to women avoiding or delaying accessing health [[20](#_ENREF_20) [21](#_ENREF_21)], and avoiding confrontation about humiliating treatment due to fear of jeopardising maternity care [[27](#_ENREF_27)]. Pregnant women also feel that they receive inadequate information about nutrition and physical activity from health professionals, and are often left confused by conflicting information [[28](#_ENREF_28)].

Midwives have expressed the need for training and skills development to address the difficulties faced in their clinical practice which lead to inequalities in the support they provide for obese women, comparing obesity with other complex sensitive topics for which structured training is available (e.g. domestic violence) [[19-21](#_ENREF_19)]. NICE have identified professional development as a priority area in relation to maternal obesity, recommending that health professionals should have knowledge and skills to advise on weight management (including weight related behaviours), behaviour change, sensitive communication techniques, and knowledge of local services [[15](#_ENREF_15) [29](#_ENREF_29)]. Although these recently published guidelines specific to weight management in pregnancy are now in the public domain and therefore available to midwives, passive dissemination of clinical guidelines is an ineffective means of implementation into clinical practice (and is therefore likely to reduce the chance of positive health outcomes for patients), and effective implementation requires more active strategies [[30](#_ENREF_30) [31](#_ENREF_31)]. While recognising the remit of their public health role, midwives identify a key barrier to addressing obesity and weight management in pregnancy to be a lack of knowledge, skills and confidence to do this effectively, leading to inequalities in the advice and support offered between midwives and maternity units [[1](#_ENREF_1) [22](#_ENREF_22)]. This potentially misses the best available life-course opportunity to engage women with weight related behaviour change.

***7.2 Specific objectives or hypotheses to be addressed by this pilot trial:***

Overall research question (to be addressed by a future definitive trial):

1. Does a theory-based intervention facilitate the implementation of weight management guidelines into midwifery practice?
2. Does midwifery implementation of weight management guidelines mediate obese pregnant and postnatal women’s weight and weight-related behaviours?

Pilot RCT aim:

To pilot a theory-based intervention to facilitate the implementation of weight management guidelines into midwifery practice

Pilot study objectives:

1. Pilot the intervention delivery, data collection and analysis methods to ascertain feasibility and acceptability thereof
2. Identify the intervention’s active ingredients (in success or failure) through process evaluation
3. Collect baseline and outcome data required to inform sample size estimations and scope data collection procedures for economic evaluation within the definitive trial

***7.3 Intervention development (including existing evidence-base and theoretical underpinning):***

The intervention development used a systematic approach recommended for the development of interventions targeting health professionals [[32](#_ENREF_32)].

The NICE guideline weight management recommendations [[15](#_ENREF_15)] were adapted using the TACT (Target, Action, Context, Time, [[33](#_ENREF_33)]) framework to explicitly define the behaviours within the guideline recommendations. The defined behaviours were grouped into six behaviour areas based on similarity (figure 1). The process of the behaviours being carried out by a midwife in a routine consultation (e.g. booking appointment) was mapped out, and the behaviours were further grouped into two overarching themes for intervention (1. Weight communication behaviours; 2. Weight management behaviours, figure 1).

*Figure 1. Grouping and process of NICE guideline recommended behaviours [*[*15*](#_ENREF_15)*]*

**2. Weight management behaviours**

**1. Weight communication behaviours**

A systematic review was carried out to identify the barriers and facilitators (determinants) of health professionals’ weight communication and weight management behaviours in clinical practice [[19](#_ENREF_19)]. The review used the theoretical domains framework to analyse the data by applying psychological explanations and theoretical constructs of behaviour change to the evidence-base in order to identify an evidence-based theoretical basis for the intervention [[30](#_ENREF_30)]. The determinants of health professionals’ behaviours identified in the systematic review best fit with the Social Cognitive Theory (SCT) [[34](#_ENREF_34)], and therefore this is the theoretical basis of the intervention. SCT is based on the principles that the person, environment, and behaviour all interact and influence one another, and that behaviours are directly related to an individual’s behavioural goals [[34](#_ENREF_34)]. The theoretical construct at the core of the personal factors in the SCT is self-efficacy; additional constructs include outcome expectancies and goals. Within the environmental factors lies socio-cultural facilitators and impediments. There can also be behavioural determinants outside these core constructs which impact on them: in the systematic review we identified that knowledge and attitudes influenced the personal constructs of self-efficacy, outcome expectancies and goals, and therefore these have been added to the theoretical model for this intervention (figure 2; see appendices 1 and 2 for the full models for weight communication and weight management behaviours summarising the behaviours and key determinants for each construct).

*Figure 2. Social cognitive theory model for the intervention*


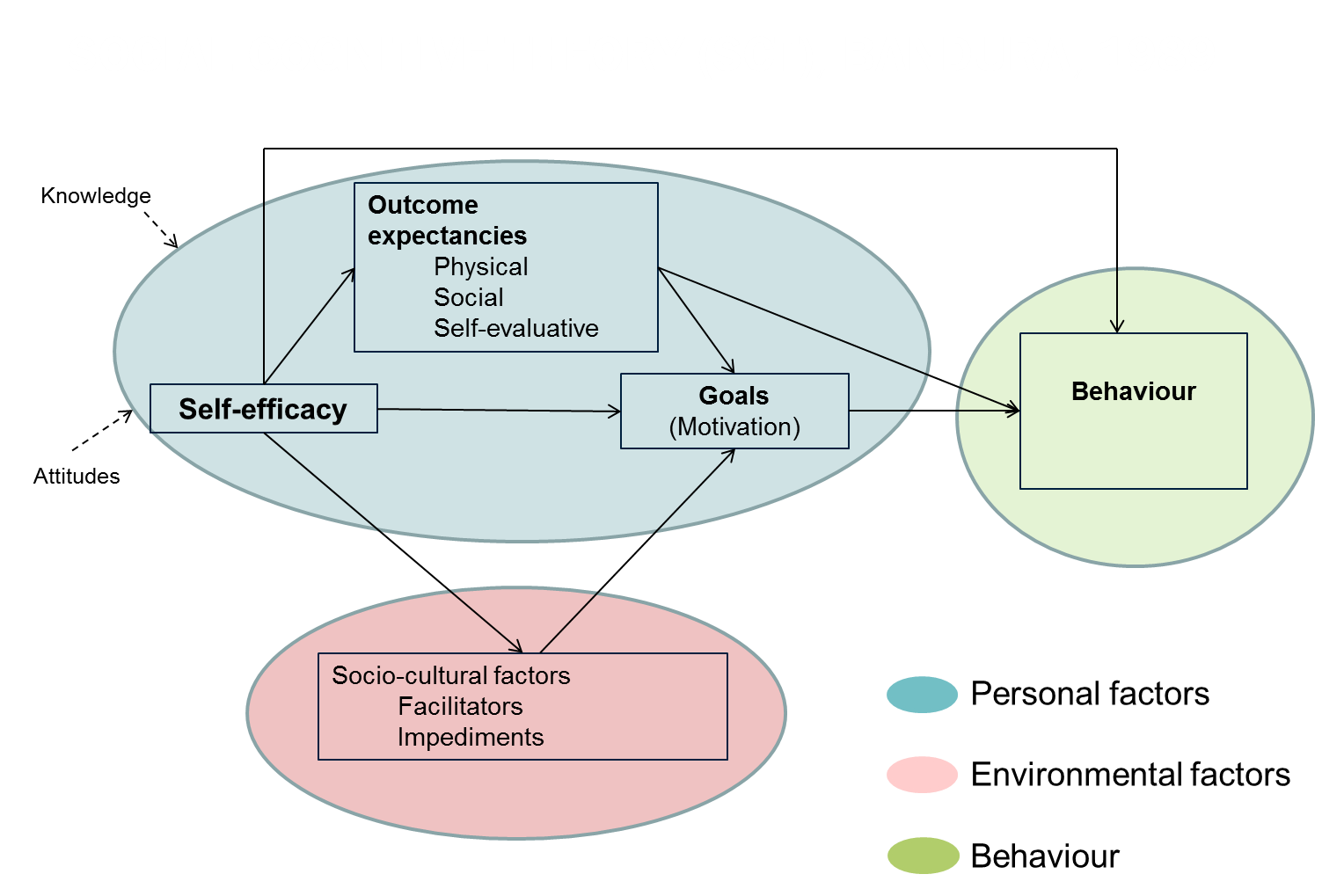


A systematic review was also carried out to identify any existing published or ongoing trials aiming to change health professionals’ maternal obesity or weight management in pregnancy behaviours. The aim of the review was to identify the effectiveness of intervention methods, behaviour change techniques and modes of delivery [[35](#_ENREF_35)]. However, this was an empty review and therefore did not identify any directly comparable studies. In the absence of this information, alternative related evidence-sources were used. These included the methods aligned with SCT [[36](#_ENREF_36)]; evidence on mode of delivery and behaviour change techniques identified in our first systematic review [[19](#_ENREF_19)]; evidence from nine related EPOC Cochrane reviews [[37-45](#_ENREF_37)]; and existing behaviour change technique taxonomies [[46](#_ENREF_46) [47](#_ENREF_47)] (table 1). This related evidence-base was incorporated into the intervention development.

*Table 1: Sources of evidence of methods, behaviour change techniques and modes of delivery for the intervention development*

| **Social Cognitive Theory** [[36](#_ENREF_36)] | **Systematic Review** [[19](#_ENREF_19)] | **9 Cochrane reviews (EPOC group)** [[37-45](#_ENREF_37)] | **Behaviour change technique taxonomies**  [[46](#_ENREF_46) [47](#_ENREF_47)] |
| --- | --- | --- | --- |
| Relevant methods:   - Enactive mastery experiences - Vicarious experiences (modeling) - Verbal persuasion and allied types of social influences - Enhancement or reduction of physiological and affective states - Facilitation | Relevant results:   - Need for specific training - Training is interactive - Include feedback from women - Preferred learning from peers - Need for comparison of behaviour with that of their peers - Required national priority, guidance and support of local practice – resources | Relevant results from the EPOC reviews:   - Use of prompts/reminders/cues was found effective - Printed educational materials - Didactic + interactive activities together were most effective, ideally with more than 1 active element - Games improve enjoyment of the intervention - Interventions tailored to prospectively identified barriers - Use of guidelines, lectures and opinion leaders | Relevant taxonomies:   - From theory to intervention: mapping theoretically derived behavioural determinants to behaviour change techniques. - A refined taxonomy of behaviour change techniques to help people change their physical activity and healthy eating behaviours: The CALO-RE taxonomy. |

Using these evidence-based information sources, the proposed intervention will consist of a one day, small group, intensive training for midwives on weight communication and weight management behaviours.

***7.4 Risks & benefits of proposed intervention:***

Risks of the intervention are minimal as the intervention aims to support midwives implement national recommendations for weight management in pregnancy into their routine practice. If successful, the outcome would be increased evidence-based practice which should result in best outcomes for the pregnant women and their babies, as well as improved midwifery confidence, skills, and knowledge through attending the training sessions.

However, the midwives will have to take one day out of their clinical practice which could result in a backlog of workload if the NHS Trusts do not provide adequate backfill. There may be burden to participants relating to attending the intervention and data collection procedures.

Potential benefits relate to midwives adherence to best evidence-based practice guidelines if the intervention is successful. This should also benefit the pregnant women who receive care from midwives in the intervention arm of the pilot trial.

***7.5 Population to be studied***

Community midwives are the primary population (see section 10 for detail). Community midwives have been specifically selected due to the majority of the NICE recommendations being related to the booking appointment (first antenatal appointment with a health professional) [[15](#_ENREF_15)]; it is the role of the community midwife to carry out this consultation. However, some NHS Trusts also have hospital-based midwives with a specific maternal obesity or weight management role (e.g. specialist public health midwives, midwives working within antenatal obesity clinics etc), and therefore these midwives should also be following the NICE weight management guidelines. Therefore any relevant hospital based midwives with a specific role to these guidelines will be invited to attend the intervention to minimise the provision of conflicting information to pregnant women.

The secondary population to be studied are pregnant and postnatal women with pre-pregnancy obesity (BMI>30kg/m^2^ identified during the booking appointment). The research intervention (training) will not involve pregnant and postnatal women. However, if the intervention is successful in changing midwifery practice then this could impact on the care that pregnant and postnatal women receive. Any change in practice should not impact on the intensity of care in the intervention group compared with controls. Any change in practice as a result of the intervention should only impact on the communication approaches of midwives, and the type of information and advice provided relating to weight management in pregnancy. Theoretically, successful implementation of the guidelines into practice should support women with their weight and weight-related behaviours. Therefore women will only be included as a secondary population for the purposes of piloting the outcome data collection measures, and to participate in the process evaluation.

# Objectives

The aim of a definitive evaluation of the proposed intervention would be to address the following research questions:

1. Does a theory-based intervention facilitate the implementation of weight management guidelines into midwifery practice?
2. Does midwifery implementation of weight management guidelines mediate obese pregnant and postnatal women’s weight and weight-related behaviours?

The primary outcome for this type of implementation intervention would relate to measures of health professionals’ practice. Secondary outcome measures would relate to measures of women’s weight status and weight-related behaviours. Such an evaluation would require a large-scale cluster RCT. Large scale evaluations of interventions can be limited by poor recruitment and retention rates, limited acceptability of and compliance with the intervention (by those delivering and receiving it), and process issues in the intervention delivery [[48](#_ENREF_48)]. The MRC stresses the importance of conducting pilot studies prior to large scale trials when the intervention has multiple components [[48](#_ENREF_48)]. Pilot studies can explore such potential issues to inform the design and conduct of a definitive study [[49](#_ENREF_49) [50](#_ENREF_50)]. Therefore a necessary prerequisite, and the focus of this study, is a pilot study to ensure methodological rigour, scientific validity, and inform the development of a protocol for that definitive trial [[49](#_ENREF_49)]. A rehearsal pilot trial of the intervention will be carried out, with integrated process evaluation. A key priority for assessment of complex interventions includes identifying how it works in everyday practice, which will be achieved through the process evaluation element of the rehearsal pilot trial [[48](#_ENREF_48)]. The aim of the proposed multi-centre rehearsal pilot cluster RCT is:

- To pilot a theory-based intervention to facilitate the implementation of weight management guidelines into midwifery practice

The specific pilot study objectives are to:

1. Pilot the intervention delivery, data collection and analysis methods to assess feasibility and acceptability thereof
2. Explore the intervention’s active ingredients (in success or failure) through process evaluation
3. Collect baseline and outcome data required to inform sample size estimations and scope data collection procedures for economic evaluation within the definitive trial

# Study Design

This is a multi-centre cluster randomised controlled parallel group trial comparing the delivery of a theory-based behaviour change intervention for community midwives versus usual practice. A cluster design is the gold standard in implementation research due to the significant risk of contamination between control and intervention groups when randomising at individual participant level [[51](#_ENREF_51)]. This is a pilot RCT and is not expected to produce definitive results. A definitive trial will be designed to evaluate the intervention fully based on the results of the pilot study.

9.1 Outcomes:

The main outcomes of the pilot study relate to whether the intervention and trial procedures are feasible and acceptable to participants, to assess the feasibility of collecting the outcome measures required for a definitive trial, and to prioritise which outcomes should be primary or secondary outcomes for a definitive trial. The outcomes for this pilot study are detailed below according to the pilot study objectives.

*9.1.1 Objective 1: Pilot the intervention delivery, data collection and analysis methods*

a) Piloting intervention delivery (training sessions for midwives) will include an assessment of fidelity of delivery, whether it is feasible to deliver the training sessions to all community midwives in the intervention arm, the number of sessions required to ensure all community midwives have an opportunity to attend the training session, the time taken to deliver the intervention (including the time taken to deliver the individual components/sessions within the training day, the total duration of the training day including breaks, and the number of weeks/months required to run the intervention completely from start to finish at each site), and any other factors which may be required for the organisation and delivery of a definitive trial (e.g. do locations in the community or in the maternity units have the best attendance rates, have all equipment and resources required for the intervention been identified, how accurate are the estimates for printing requirements for training packs?). Data collection will involve the following:

- Observation of the intervention delivery (data collection/observation by the CI)
- Video recording of the intervention delivery (data collection by the midwife facilitator (Catherine McParlin), or observer if present)
- Intervention delivery log:
  - Total number of participants eligible for the intervention for each site (data collection by local PIs/research midwives at each trust)
  - Total number of participants who attend the intervention at each site (log to be maintained by the midwife facilitator for each intervention delivery session, to include number of participants in attendance at each session)
  - Location of intervention delivery for each session (log to be maintained by the midwife facilitator for each intervention delivery session, to include location)
  - Number of intervention sessions delivered (log to be maintained by the midwife facilitator for each intervention delivery session, each session to be numbered with unique ID)
  - Dates and times of each intervention session at each site (log to be maintained by the midwife facilitator for each intervention delivery session, to include date of session)

b) Piloting data collection procedures will include recruitment and retention of participants completing outcome measures, and data yield.

- Recruitment and retention: data will be collected on eligibility and consent rates, and retention of participants providing outcome measures at the scheduled time points. Recruitment and retention data will be collected at the levels of NHS Trust, midwives, and women. Data will be analysed to identify any systematic differences between those who agree to participate in the study and the general maternity population in the NHS Trusts recruited and thereby ascertain the extent of any participation bias. Data collection will include:
  - Number of midwives eligible to be approached to provide quantitative or qualitative data (data to be collected by the by local PIs/research midwives during recruitment process)
  - Number of pregnant women eligible to be approached to provide quantitative baseline data (data to be collected by the by local PIs/research midwives during recruitment process)
  - Number of pregnant women eligible to be approached to provide quantitative outcome data and qualitative process evaluation data (data to be collected by the by local PIs/research midwives during recruitment process)
  - Numbers of NHS Trusts, midwives and women approached/accepted/declined to participate in the study (data to be collected by the by local PIs/research midwives during recruitment process)
  - Number of NHS Trusts retained until the end of the study (log of recruitment and retention to be maintained by the CI)
  - Number of midwives retained/data provided at each data collection time point until the end of the study (log of recruitment and retention to be maintained by the CI)
  - Number of women retained/data provided at each data collection time point until the end of the study (log of recruitment and retention to be maintained by the CI)
  - Characteristics of women eligible/recruited to ascertain the extent of any participant bias (recruited women will be asked to provide this information on a questionnaire; the local PIs/research midwives will have access to patient data from the electronic patient record system to provide anonymised pooled data for the recruitment time period for comparison)
- Data yield and data quality: information on data yield and quality will be used to identify any data collection methods which require further exploration (as part of the process evaluation), to refine the data collection tools and procedures where required (e.g. refine any questions that are confusing and therefore inconsistently complete in questionnaires, or change the time point for data collection if one time point results in consistently low yield or quality), and to inform decisions about what the primary and secondary outcomes should be for the definitive trial.
  - Data yield will be calculated by determining the percent of participants from whom data are collected at each time point (e.g. the percent of participants returning completed questionnaires; CI to assess)
  - Data quality will be descriptively assessed by determining the completeness (item response rates) and consistency of data for each participant at each time point, and over the duration of the study where data collection of the same outcome measures is required at multiple time points (e.g. number of questionnaire questions completed, consistency (where relevant) of specific questions being completed; CI to assess)

c) Piloting data collection for the planned analysis of a definitive trial will be carried out to ensure that the data being collected using the outcome measures are appropriate and sufficient. Additionally, the time and procedures required for data manipulation will be recorded to plan this workload for the definitive trial. This includes any data manipulation (e.g. coding) required to be carried out by the CI/statistician and by the local PIs/research midwives within the NHS Trusts (e.g. anonymising identifiable data). Data collection will include:

- Statistician perspectives on the data provided for analyses (e.g. format, limitations, additional data required not previously anticipated) (to be discussed with the CI, statistician and co-investigators)
- Time/procedures record for data manipulation (to be maintained by the CI, statistician, and local PIs/research midwives for each site)

*9.1.2 Objective 2. Explore the intervention’s active ingredients (success or failure) through process evaluation*

The primary purpose of the process evaluation is to identify if the intervention can be refined and whether trial procedures are feasible and acceptable to participants (e.g: Are there key elements missing from the intervention? Does the intervention address the main difficulties in providing weight management advice in routine consultations? Is the advice and support from midwives acceptable to pregnant women?). The process evaluation also aims to provide insight into intervention failure or unexpected outcomes; intervention success and how to optimise it; clarify causal mechanisms; and assess feasibility and acceptability of the trial procedures to midwives and pregnant women (e.g. burden of data collection). Therefore the process evaluation will be carried out for: i) the content, resources and delivery of the intervention (training sessions), ii) the implementation of guidelines into routine midwifery practice following the intervention, and iii) among women with obesity receiving midwifery care during pregnancy (to be followed up postnatally). The process evaluation will also be informed by objective 1b to explore any issues arising on the clarity or appropriateness of the data collection tools developed (e.g. if questionnaires consistently not completed), and the processes of data collection to ensure it is comprehensive (e.g. optimum gestation/postnatal time points to weigh women and send questionnaires).

i) Process evaluation for the content, resources and delivery of the intervention will include assessment of how midwives respond to the intervention delivery (e.g. format of the day, timing of sessions), content scope and level of detail, if any aspects were particularly confusing/unclear or well received, and perspectives of the resources provided. Data collection will involve:

- - Observation of midwives’ response to the intervention delivery and content (data collection/observation by the CI)
  - Debriefing interviews with the midwife facilitator after each intervention delivery session (debrief to be carried out by the CI and notes recorded in an intervention delivery diary)
  - Evaluation forms (including a combination of rating scales and open questions) to be completed by all the midwives attending the intervention (anonymous evaluation forms to be distributed by the midwife facilitator at the start of the intervention session, and collected using a “post box” at the end of the intervention session to maintain participants confidentiality as they may wish to include comments about the midwife facilitator/observer on the evaluation form)
  - Focus groups with midwives who have received the intervention will explore acceptability of the intervention, and their experiences and views relating to the intervention delivery, materials, and resources. Questions will be developed based on the results of the observations, debriefing interviews and evaluation forms to further explore midwives perspectives of the intervention content and delivery (data collection to be carried out by the CI)

ii) Process evaluation for the implementation of guidelines into routine midwifery practice will include a combination of quantitative and qualitative methods to assess the mechanism of behaviour change, and the feasibility and acceptability of data collection procedures.

- Quantitative: Implementation interventions require quantitative process variables of the determinants of midwives behaviours to be measured in order to identify the causal mechanisms of behaviour change. As this is a pilot study, the purpose of performing this quantitative process evaluation is to test the feasibility and acceptability of the data collection procedures (as with objective 1b), and the burden of data collection. The quantitative process evaluation measures will be questionnaire-based and aligned to the behavioural determinants included in the SCT model being used in the intervention (SCT questionnaires to be distributed by the local PIs/research midwives in each site with SAE for return of the questionnaires to the CI):
- Qualitative: The qualitative process evaluation will utilise the focus groups with midwives who have received the intervention (same focus groups as described in 9.1.2.i, to be carried out by the CI) to explore the ease implementing the training into their practice.

iii) Process evaluation for women with obesity receiving midwifery care during pregnancy will involve semi-structured interviews during pregnancy and at 6 months postpartum (data collection to be carried out by the CI, and Project Research Midwife Catherine McParlin).

Interviews will explore aspects of the intervention which require refining based on the results of objective 1b (relating to data collection procedures and tools), as well as women’s experiences of the advice and support provided by their midwives, and any barriers of facilitators to women being able to implement the midwives advice into their own behaviours.

*9.1.3 Objective 3. Collect data required to inform sample size estimations and scope data collection procedures for economic evaluation within the definitive trial*

i) Sample size estimations: The data on intervention primary and secondary outcome measures for the midwives and women, and rates of attrition, will be used to estimate sample size requirements for a definitive trial (see section 16). Estimating the sample size required for a definitive trial will be carried out by the trial statistician.

ii) Scope data collection procedures for economic evaluation: The basic framework of the potential outcomes that may follow from the midwife training intervention, and the hypothesised causal links are presented in appendix 3. Correspondingly, appendix 4 lists the anticipated cost and outcome information relevant to a definitive trial and economic modelling including: the measures that will aim to be recorded in the different phases of the pilot trial; which outcomes would need to be modelled; and how and when the data from the trial will be collected. Long term outcomes, such as maternal and child obesity, will not be recorded within the time frame of the pilot study (or a subsequent definitive trial), and these will have to be modelled using observational data obtained from the literature. Appendix 4 also incorporates key trial outcomes that measure the impact of the intervention, which are intermediate outcomes from the perspective of economic evaluation. These outcomes include the constructs representing the SCT (i.e. midwives determinants of behaviour: self-efficacy, outcome expectancies, intentions, knowledge and attitudes), midwife clinical practice behaviours, and pregnant/postnatal women’s diet and exercise behaviours. These are essential intermediate outcomes that help explain the link between intervention and health outcomes. These outcomes theoretically enable improved health outcomes, such as the well-being of pregnant/postnatal women, reduced work-related stress for midwives, reduced complications for pregnant women, improved long-term outcomes for pregnant women and their babies. A hypothesised balance sheet of the costs and benefits relevant to a cost-effectiveness analysis of the intervention is presented in appendix 5. The pilot study will assess the feasibility of measuring the well-being of pregnant women using an appropriate well-being assessment instrument at the relevant time points.

There are two phases required in the pilot study to scope the data collection requirements and procedures for economic evaluation in a definitive trial:

Phase 1. Identification of data requirements for an economic evaluation case report form: Data collected throughout the pilot study will be used to update the information in appendices 3-5 to inform the data requirements for full economic evaluation in a definitive trial. This will include any unexpected costs and benefits of the intervention identified by midwives and women throughout the questionnaire and qualitative data collection. A random sample of 18 of medical records (i.e. women consenting to provide baseline data) will be obtained from each participating site to identify the type of data routinely recorded which can be used to develop specific data items required for a case report form for economic evaluation.

Phase 2. The feasibility of data collection using case report forms: An audit will be carried out using the medical records of women recruited and consenting to provide quantitative outcome data in each participating site to test the feasibility of extracting the case report form data items from routine data sources. The feasibility of extracting routine data on child growth from the child’s “red book” health records up to one year postnatal will be tested (these are the child’s health and vaccination records held by the parent which includes growth chart from the weights taken by health visitors). Phase 2 will identify which data items can be retrieved from routine data sources, and which data items will require additional data collection for a definitive trial.

9.2 Definition of end of study:

The end of study will be the last participant’s final study contact. This will comprise the last pregnant woman’s 12 months postnatal follow up.

# Participants

The intervention and control participants will be community midwives, and any hospital-based midwives in the pilot sites with a specific maternal obesity or weight management role (e.g. public health midwife). The intervention aims to recruit all eligible midwives in the intervention arm to receive the intervention. This is due to the nature of shared care between community midwives meaning that any one pregnant woman might see a different community midwife from the team at each antenatal visit. If all midwives receive the intervention, this should increase consistency of advice and support for women who may receive care from a team of midwives. All midwives receiving the intervention will be asked to complete evaluation forms as part of the process evaluation. A further random subgroup of midwives from all participating Trusts will be selected to provide specific outcome and process evaluation data for the pilot. The study setting will be in the community where midwives practice. The intervention will be delivered in a training environment dependent on local availability for the pilot site communities (e.g. hospital academic centre, community NHS training facilities). The pilot study will take place in the North East of England.

Outcome measures will also be collected from eligible pregnant women (followed up postnatal), although these women will not receive the research intervention (midwifery training). For the purposes of this study, these women are also considered to be participants.

10.1 Inclusion criteria

- Midwives:
  - Midwives who have provided written informed consent for participation in the study prior to any study specific procedures
  - Community midwife role
  - Hospital-based midwife with a specific maternal obesity or weight management role
- Pregnant women:
  - Pregnant women who have provided written informed consent for participation in the study prior to any study specific procedures
  - Booking body mass index ≥30kg/m^2^ (proxy measure for pre-pregnancy BMI)
  - Age ≥ 18 years (teenage pregnancies require specific nutritional support)
  - Singleton pregnancies (multiple pregnancies require specific nutritional support)
  - Women who have had their 12 week ultrasound scan and their pregnancy has progressed beyond the high risk period for miscarriage

10.2 Exclusion criteria

- Midwives:
  - Hospital-based midwives without a specific maternal obesity or weight management role
  - Non-midwifery health professionals
- Pregnant women
  - Pregnant women with a medical condition other than obesity which requires them to receive specialist weight management advice for that condition (e.g. women with pre-gestational diabetes who are attending a specialist antenatal diabetes clinic, pregnant women who have had bariatric surgery (e.g. gastric band or bypass) and require specialist nutritional support)
  - Pregnant women with substance misuse or known cause for concern (e.g. domestic violence)

- Inability to speak/read English is an exclusion criteria for both midwives (to whom it is not expected to apply) and pregnant women. This is due to the questionnaire nature of the data collection and lack of validation in non-English language. Further feasibility work will be carried out with NHS Trusts who provide antenatal care for a high proportion of women from Black and Minority Ethnic (BME) Groups to assess transferability of the intervention to populations with more varied ethnic diversity than the North East of England to further inform intervention development prior to a definitive trial. This feasibility work will require a stand-alone protocol and will be subject to a separate ethics application.

# Screening, Recruitment and Consent

Screening, recruitment and consent procedures will be required for midwives receiving the research intervention, and midwives and pregnant women providing quantitative and qualitative outcome and process evaluation data.

11.1 Identification and screening of midwife research participants

- Potential midwife participants will be identified through staff lists at the participating sites by the local PIs/research midwives who have access to staff data.
- An eligibility screening form will be completed by the local PIs/research midwives to document participants’ fulfilment of the entry criteria for all midwives considered for the study and subsequently included or excluded.

11.2 Recruitment procedures for midwife research participants

- All potential participants eligible to receive the intervention (training) will be invited to participate and the study explained to them by the local PIs/research midwives within the intervention arm NHS Trusts. A study Participant Information Sheet (PIS) for participating in the intervention will be provided at this time and the midwife will be allowed to take this away for consideration.
- We aim to recruit 15 midwives in each participating NHS Trust to provide quantitative outcome data. To allow for withdrawal and loss to follow up, a random selection of 18 midwives in each site will be invited to provide quantitative data for the study (including baseline data, outcome measures, and quantitative process evaluation measures) and the study explained to them by the local PIs/research midwives within the NHS Trusts. A study PIS for collecting outcome data will be provided at this time and the midwife will be allowed to take this away for consideration.
- We aim to recruit a further 5 midwives from each NHS Trust to provide qualitative process evaluation data. To allow for withdrawal and loss to follow up, a random selection of 7 midwives in each site will be invited to participate in qualitative focus groups and the study explained to them by the local PIs/research midwives within the NHS Trusts. Random sampling has been chosen for this element of qualitative research as an attempt to avoid engaging with only those midwives with a specific interest in the topic, as the process evaluation requires the exploration of positive and negative perspectives. A study PIS for focus groups will be provided at this time and the midwife will be allowed to take this away for consideration.
- A screening log will be kept by the local PIs/research midwives in each site to:
  - Document details of midwives invited to participate in the intervention, to provide quantitative data, and to provide qualitative focus group data.
  - Document reasons for non-participation among midwives who decline participation of any stage if they are happy to provide this information.
  - Ensure potential participants are only approached once for each aspect of recruitment (i.e. the same midwives will only be approached once to receive the intervention, and among those who agree midwives will only be approached once to provide quantitative or qualitative data).
- Recruitment to provide the quantitative baseline (and follow up) data will be carried out prior to randomisation of clusters into intervention or control arms. Midwife participants to be recruited to receive the intervention, and for qualitative focus groups, will only be required in the intervention arm. Therefore the recruitment for these stages will take place following randomisation of clusters.
- Midwives will be reimbursed any travel expenses incurred relating to recruitment, participating in the intervention, and data collection.

11.3 Consent procedures for midwife research participants

- Informed consent discussions will be untaken by a member of the research team (as per delegation log) involved in the study, with opportunity for midwives to ask any questions.
- Following receipt of information about the study, midwives will be given reasonable time to decide whether or not they would like to participate.
- Those wishing to take part will provide written informed consent by signing and dating the relevant study consent form which will be witnessed and dated by a member of the research team with documented, delegated responsibility to do so.
- Written informed consent will be obtained prior to any study specific procedures/investigations.
- The original signed consent form will be retained in the Investigator Site File, and a copy provided to the midwife participant.
- The right to refuse to participate without giving reasons will be respected.
- 1^st^ consent: Consent will be taken from the midwives randomised to provide quantitative outcome measures prior to baseline data collection, and prior to intervention delivery.
  - Due to the geographical location of some community midwife bases, face-to-face consent is not practical. An alternative option for the midwives questionnaire-based data collection is for the PIS and questionnaires to be posted to community midwives. The community midwives have the option of discussing the research over the phone with a member of the research team, and completion and return of the questionnaire is implied consent.
- 2^nd^ consent: Consent will be taken from all the midwives in the intervention arm to receive the intervention prior to attending the intervention.
- 3^rd^ consent: Consent will be taken from midwives recruited to participate in focus groups following receipt of the intervention.

11.4 Identification and screening of women to provide outcome and process data

- Screening for eligible pregnant women participants will use the antenatal booking data recorded on the participating sites electronic patient records for the recruitment time period to assess eligibility of women attending their 12 week scan appointment. Screening will be carried out by the local PIs/research midwives who have access to this data at their local site.
- An eligibility screening form will be completed by the local PIs/research midwives to document participants’ fulfilment of the entry criteria for all women considered for the study and subsequently included or excluded.

11.5 Recruitment procedures for women to provide outcome and process data

- Baseline data: We aim to recruit 15 pregnant women in each NHS Trust to provide quantitative baseline data. To allow for withdrawal and loss to follow up, a random sample of 18 eligible women attending the NHS Trust for their 20 week scan at each site will be invited to provide quantitative questionnaire data at baseline, prior to the intervention delivery in order to identify the similarity of outcome measures within and between sites prior to the intervention. Recruitment will be carried out by the local PIs/research midwives and the study will be explained to potential participants. A study PIS for one-off collection of baseline data at 20 weeks gestation will be provided at this time and the women will be allowed to take this away for consideration. Recruitment for women providing baseline data will be carried out prior to randomisation of clusters into intervention or control arms.
- Outcome data: We aim to recruit 15 pregnant women in each NHS Trust to provide quantitative outcome data. To allow for withdrawal and loss to follow up, women at each site will be approached and invited to provide quantitative post-intervention outcome data (questionnaire-based data following their 12 week scan; weight measurements and questionnaire-based data in the 3^rd^ trimester; and repeat measures postnatally) until 30 women per site have consented and returned their first questionnaire. The study will be explained to all women approached by the local PIs/research midwives. A study PIS will be provided at this time and the women will be allowed to take this away for consideration.
- Qualitative data: we aim to recruit and interview 5 pregnant women from each NHS Trust to provide qualitative process evaluation data (semi-structured interviews) in their 3^rd^ trimester and again at 3 months postnatal. To allow for withdrawal and loss to follow up, the opportunity to participate in this qualitative research will be discussed with all women consenting to provide quantitative outcome data until 10 eligible women at each site have consented. A study PIS will be provided at this time and the women will be allowed to take this away for consideration.
- As a recruitment and retention incentive due to the potential burden of data collection without any direct benefit of participation in the study, women will receive a £10 high street gift voucher for every data collection time point (maximum of £10 for women who provide only baseline quantitative data, £60 for women who provide quantitative outcome data at all time-points, and £80 for women who provide quantitative outcome data plus qualitative interview data at both time-points). Women will also be reimbursed any travel expenses incurred relating to recruitment and data collection.
- The random sampling and recruitment procedures will continue until the target sample sizes are reached.
- A screening log will be kept by the local PIs/research midwives in each site to:
  - Document details of women invited to provide quantitative outcome data
  - Document details of women invited to provide qualitative interview data
  - Document reasons for non-participation among women who decline participation of any stage if they are happy to provide this information.
  - Ensure potential participants are only approached once for each aspect of recruitment (i.e. the same women will only be approached once to provide quantitative outcome data and to provide qualitative interview data).

11.6 Consent procedures for women to provide outcome and process data

- Informed consent discussions will be untaken by a member of the research team (as per delegation log) involved in the study, with opportunity for women to ask any questions.
- Following receipt of information about the study, women will be given reasonable time to decide whether or not they would like to participate.
- Those wishing to take part will provide written informed consent by signing and dating the relevant study consent form which will be witnessed and dated by a member of the research team with documented, delegated responsibility to do so.
- Written informed consent will be obtained prior to any data collection.
- The original signed consent form will be retained in the Investigator Site File, a copy will be filed in the women’s antenatal notes, and a copy provided to the participant.
- The right to refuse to participate without giving reasons will be respected.

# Study Intervention Details

The details of the intervention are reported using the template for intervention description and replication (TIDieR) checklist and guide [[52](#_ENREF_52)].

**Brief name:** GLOWING Pilot Cluster RCT

**Why:** The background to, and theoretical basis of, the intervention are described in section 7. The aim of the intervention and specific objectives of the pilot study are detailed in section 8.

**What: Materials and Procedures**

Materials – intervention arm only: All teaching materials will be provided to the midwives participating in the intervention in a training pack, as well as being available via a study website. Midwives will also be provided with one hard copy each of the First Steps Nutrition Trust charity resource “Eating well for pregnancy: A practical guide” (<http://www.firststepsnutrition.org/newpages/Pregnancy/pregnancy_practical_guide_for_healthy_pregnancy.html> [last accessed 23/11/2015]).

The NHS Trusts in the intervention arm will also be provided with resource packs to share with pregnant women with obesity throughout the duration of the pilot study. The resources to be included in this pack comprise of existing credible information resources on maternal obesity and weight management in pregnancy from charities and Start4Life. An additional leaflet is being developed for this study to identify local support for women with pregnancy and postnatal nutrition and physical activity (e.g. local aqua natal classes), as well as listing further credible information sources (e.g. NHS Choices, Start4Life).

- Tommy’s the Baby Charity:
  - Booklet: Managing your weight in pregnancy <https://www.tommys.org/sslpage.aspx?pid=602&nccsm=21&__nccspID=967> [last accessed 23/11/2015]
  - Leaflet: Your guide to staying active in pregnancy <http://www.tommys.org/file/Exercise.pdf> [last accessed 23/11/2015]
- Leaflet from First Steps Nutrition Trust identifying resources available for women to purchase (hard copies) or download for free, including:
  - Pregnancy: Eating well for pregnancy: A practical guide; <http://www.firststepsnutrition.org/newpages/Infants/infant_feeding.html> [last accessed 23/11/2015]
  - Postnatal: Eating well for New Mums: Including information for breastfeeding mothers; Eating well: the first year; Eating Well Recipe Book; Breastmilk and breastfeeding: a simple guide; and Infant milks: a simple guide to infant formula, follow on formula, and other infant milks <http://www.firststepsnutrition.org/newpages/Infants/infant_feeding.html> [last accessed 23/11/2015]
- Start4Life
  - Booklet: healthy habits for baby and you <https://campaignstorage.blob.core.windows.net/start4life/development/uploads/ckeditor/attachments/9/Start4Life-Healthy_Habits.pdf> [last accessed 23/11/2015]

Procedures: The intervention will be delivered over one day (see appendix 6 for overview of the day). The separate sessions will include graded tasks for weight communication behaviours in the morning (appendix 7), and for the weight management behaviours in the afternoon (appendix 8), followed by a consolidation session at the end of the day (appendix 9). The intervention will include a combination of didactic and interactive elements, and will be delivered as graded tasks throughout the intervention delivery (one day training). Graded tasks start with small tasks to gain confidence, and each subsequent task builds on the previous one.

**Who will provide:** The intervention delivery (training) will be carried out by the project research midwife (Catherine McParlin). The evidence-base on midwifery training requirements for maternal obesity management identified that it was important for the trainer to be a midwife to have understanding of their role [[21](#_ENREF_21)]. Catherine has been a registered midwife for 20 years, and a research midwife for 14 years.

Catherine has expertise in the topic area having recently completed a PhD thesis titled “Physical activity behaviour in overweight and obese pregnant women”, and working as a research midwife on the UPBEAT RCT (a national RCT of a weight management intervention for obese pregnant women - ISRCTN89971375).

In relation to facilitating midwifery training, Catherine is registered to complete online training at Newcastle University on presentation skills (<http://www.ncl.ac.uk/staffdev/lrc/personal/presentations.htm>) and body language (<http://www.ncl.ac.uk/staffdev/lrc/personal/bodylang.htm>), as well as a two day Introduction to Learning and Teaching in Higher Education (ILTHE) workshop (<http://www.ncl.ac.uk/staffdev/workshops/programmes/academic/itlhe/>). These training modules will be completed prior to the intervention delivery. Catherine will also be mentored by the CI (Nicola Heslehurst) in relation to teaching skills. NH is a lecturer with 10 years of experience teaching medical and allied health professional undergraduate and postgraduate students.

**How:** The intervention will be delivered face to face in small groups of approximately six community midwives.

**Where:** The intervention delivery will take place in community or hospital based education facilities, dependent on availability in the locality of the maternity services in the intervention arm (e.g. NHS Trust academic centres).

**When and How Much:** The intervention will last for one full day and be delivered once to each community midwife in the intervention arm.

**How well:** Fidelity of the intervention delivery (training sessions) will be explored using direct observation (NH) and video recording (see section 9). Any deviations from the planned intervention delivery will be recorded and discussed with the project research midwife (debrief) and midwifery participants (focus groups) as part of the process evaluation. This data will be used to refine the intervention delivery plan for the definitive trial (e.g. any aspects confusing, not well received, and not easy to deliver in this format?).

# Randomisation

13.1 Intervention randomisation

Computer randomisation of the four pilot clusters (NHS Trusts) into intervention or control arms will be will be stratified by size of maternity service within the NHS trust (based on number of bookings/year in each NHS Trust: large units – Newcastle and Northumbria NHS Trusts; small units - Gateshead and South Tyneside). Randomisation will be performed by a statistician using anonymised unique IDs for each trust to prevent allocation bias (e.g. Large1, Large2; Small1, Small2). Midwives will be consented to take part and baseline data will be collected before randomisation (i.e. allocation concealment prior to consent and baseline data collection).

13.2 Randomisation for data collection

The midwives and women (baseline only) invited to provide quantitative and qualitative data for the pilot study will be randomly selected within each cluster. Local PIs/research midwives with access to staff lists and electronic medical records at each site will allocate a unique anonymised ID number to each eligible participant (midwife and women). Random selection of midwives and women at each site will be carried out by a statistician using the list of anonymised unique IDs. For the follow up post-intervention data collection from pregnant women, a convenience sample will be used rather than randomisation. The ultrasound scan clinic appointment lists will be accessed by the research midwives in each site, and the women’s electronic records will be screened against the inclusion criteria to identify eligible women to approach for consent.

# Blinding

Blinding is not applicable to this study. Due to the nature of the intervention it is not possible for the intervention delivery team or midwives to be blinded. However, aspects of the study where some degree of blinding will be utilised including:

- The NHS Trusts will be given a unique ID code and the statistician performing the randomisation will be blind to the NHS Trusts during the process of cluster randomisation
- The midwives and pregnant women will be given a unique ID code and the statistician performing the randomisation will be blind to the participant details during the process of random selection of midwives and women
- The pregnant women involved in the study will not be informed by the researchers whether their midwives are in the intervention or control arm of the study, and midwives will be asked not to disclose this information to women during their consultations
- Midwives will not be aware which arm of the trial their NHS Trust has been allocated to when consenting and providing baseline data

# Study Data

In addition to the data required to meet the aims of the pilot study (detailed in section 9), one of the purposes of the pilot study is to determine which outcome to use as the primary outcome and which should be secondary in a definitive trial. The proposed primary and secondary outcomes will be collected during the pilot study in line with the primary and secondary aims of the intervention.

- Primary aim: midwives behaviours
- Primary outcome: change in midwives clinical practice
- Potential primary outcome measures:
  - Midwives self-reported practice
  - Women’s self-report of their midwives practice
  - Case note audit of recorded midwifery practice
  - Vignettes of different types of consultation (simulated practice)
- Secondary aim: women’s weight and weight-related behaviours
- Primary outcome: change in weight from booking
- Potential primary outcome measures:
  - Pregnancy weight: 3^rd^ trimester,
  - Postnatal weight: 3, 6, 9, 12 months
- Secondary outcomes: 3^rd^ trimester and 3, 6, 9, 12 months postnatal
  - Dietary behaviours
  - Physical activity behaviours
  - Psychosocial measures for weight-related behaviours
  - Therapeutic alliance with midwives (3^rd^ trimester of pregnancy only)
  - Quality of life

All primary data collection will be anonymised using the participant’s unique study ID number. An overview of the study data are presented in table 2.

Table 2. Data collection type, populations, sites, time points, and consent

|  | **Baseline (pre-intervention)** | **Intervention delivery** | **1 month post intervention** | **3 months following intervention delivery (to all midwives)** | **6 months following intervention delivery (to all midwives)** | **3 months postnatal** | **6 months postnatal** | **9 months postnatal** | **12 months postnatal** |
| --- | --- | --- | --- | --- | --- | --- | --- | --- | --- |
| **Maternity electronic patient records (all clusters)** | Eligible pregnant women’s characteristics |  |  |  |  |  |  |  |  |
| **Random sample of 18 women attending 20 week scan/ cluster (all clusters)** | Consent, women's characteristics, questionnaire data.  Case note audit (economic evaluation phase 1) |  |  |  |  |  |  |  |  |
| **Random sample of 18 midwives/ cluster (all clusters)** | Consent, midwives characteristics, questionnaire data |  |  | Questionnaire data | Questionnaire data |  |  |  |  |
| **All community midwives/cluster (intervention arm, 2 clusters)** |  | Consent, intervention delivery, completion of evaluation forms |  |  |  |  |  |  |  |
| **Random sample of 7 midwives/ cluster (intervention arm, 2 clusters)** |  |  | Consent, focus groups |  |  |  |  |  |  |
| **Convenience sample of 30 women/ cluster (all clusters)** |  |  |  |  | Consent women at 12 week scan and questionnaire data; follow up at 36 weeks, questionnaire data and weight measurement | Questionnaire data, weight measurement, case note audit | Questionnaire data, weight measurement | Questionnaire data, weight measurement | Questionnaire data, weight measurement |
| **Convenience sample of 10 recruited women/cluster (all clusters)** |  |  |  |  | Consent at 12 weeks, semi-structured interviews (3rd trimester) |  | Semi-structured interviews |  |  |

Study data collection details:

- Maternity electronic patient records: pregnant women’s characteristics including maternal age, BMI, ethnic group, parity, employment status, socio-economic status (determined by postcode anonymised by data linkage with index of multiple derivation data) will be retrieved from the electronic patient records for all eligible women in their 3^rd^ trimester during the recruitment period. Data will be anonymised and aggregated to compare with recruited women to determine any participation bias for each site.
- Women at 20 week scan – baseline only: eligible women will be approached and consented by a member of the research team (as per delegation log) before the intervention delivery to provide baseline data on their characteristics (including maternal age, gestation, BMI, ethnic group, parity, employment status, qualifications, postcode), questionnaire data. This is a one off data collection to compare how similar women are within and between sites before the midwives caring for them have received any intervention. The questionnaire data are:
  - Self-report of their midwives practice specific to the guideline recommendations (developed to reflect the midwives self-reported routine practice behaviour measurements)
  - Dietary Quality Score (DQS – validated in non-pregnancy with additional pregnancy and postnatal specific questions included)
  - Pregnancy Physical Activity Questionnaire (PPAQ – validated in pregnancy)
  - Psychosocial measures for understanding weight-related behaviours in pregnant women questionnaire (validated in pregnancy)
  - Therapeutic alliance with midwives (Health Care Alliance Questionnaire - validated in pregnancy)
  - Quality of life (EQ5D 5L)
- Case note audit to inform economic evaluation: the medical records of women consenting to provide baseline data prior to intervention delivery to inform the data collection requirements for economic evaluation (phase 1 as detailed in section 9.1.3).
- Midwives: Midwives will be consented at baseline, and asked to complete a form with their characteristics (number of years practice, speciality, ethnic group, age, and gender), and questionnaire data. The questionnaire data collection will be repeated at two further time points post-intervention. The questionnaire will include:
  - Self-reported routine practice specific to the guideline recommendations (current weight communication and weight management clinical practice/behaviours)
  - SCT constructs: self-efficacy, outcome expectancies, goals/intentions (weight communication and weight management specific),
  - Additional constructs identified in the SCT model: knowledge of the guideline recommendations and attitudes towards obese populations
  - Vignettes of different types of consultations (based on the type of consultations midwives report as challenging in the existing evidence-base)
- Eligible midwives in the intervention arm: Midwives will be consented to receive the intervention (including observations and video recording), and will be asked to complete intervention evaluation forms
- Eligible midwives in the intervention arm: Midwives in the intervention arm will be consented to participate in focus groups as part of the process evaluation
- Eligible women at 12 week scan appointment: eligible women will be approached and consented to provide data on their characteristics (including maternal age, gestation, BMI, ethnic group, parity, employment status, postcode), questionnaire data, and 3^rd^ trimester weight measurements (to calculate weight change from booking). Some of the questionnaire data and weight measurements will be repeated postnatally (see table 2 for details of pregnancy and postnatal specific data collection). Recruited and consenting women’s case notes will be audited to determine feasibility of using this data source to inform the primary outcome, and to test the data collection form developed for the economic evaluation (phase 2 detailed in section 9.1.3). Case notes are retained by pregnant women antenatally and returned to the NHS Trust after discharge; therefore the audit will take place 3 months postnatally when case notes are returned.
- A subgroup of eligible women recruited to provide outcome data: women will be approached and consented by a member of the research team (as per delegation log) to take part in semi-structured interviews as part of the process evaluation. Interviews will take place during their last trimester of pregnancy, and again postnatally.

# Statistical & Analytical Considerations

All quantitative and qualitative data analysis will be descriptive with the purpose of informing the development of the definitive trial. Analysis will include fidelity of intervention delivery (i.e. have all components of the intervention been delivered as planned), any intervention organisation and delivery requirements, data collection procedures for a definitive trial, data requirements, timescale and procedures for data manipulation for a definitive trial.

Thematic analysis will be used for the qualitative data (by the CI and Project Research Midwife). The analyses will be used to inform the further development and refinement of the intervention content, delivery, and data collection procedures and tools prior to a definitive trial.

16.1 Statistical Analysis Methods

The data analysis of the outcome measures will be descriptive (percentages, means and standard deviations or 5-number summaries as appropriate), with the primary aim of providing estimates of key trial parameters, including rates of recruitment, retention, data completion, and the variability of proposed outcome measures. These key trial parameters will be used to inform power calculations for the definitive trial (see section 9.1.3), and to inform decisions about which outcome measures to use in the definitive trial (e.g. are any time points for maternal weight status consistently complete/incomplete, what data we can yield from the case note audit etc). Analyses required to inform power calculations for a definitive trial are:

- An estimate of the variability (standard deviation) for those in the control group for continuous outcomes measures
- An estimate of proportions (percentages) for categorical outcome measures
- An estimate of the intra cluster correlation (ICC) for these measures to inform the power calculation, though given the imprecision in this estimate, due to the small sample size, other sources of estimates will need to be explored.

16.2 Pilot Study Sample Size

Sample sizes for pilot studies are typically not informed by formal power calculations, but rather by pragmatism and resource constraints; it has been proposed that outcome data from approximately 30 participants per arm is adequate [[49](#_ENREF_49) [50](#_ENREF_50)]. Four clusters (NHS Trust maternity services) will be studied; two per arm.

All community midwives in intervention arm units will be targeted to receive the intervention, as shared midwifery care requires all midwives within the team to be consistent in their provision of care to this population. The aim is to obtain outcome data from 30 midwives and 30 pregnant women in each arm (15/cluster). In each cluster we will aim to recruit 18 midwives, 18 pregnant women (baseline), and 30 pregnant women (post-intervention follow up) to provide outcome data for statistical analysis in the pilot trial, to allow for withdrawal and loss to follow up. The aim is to obtain qualitative process evaluation data from 5 midwives in each NHS Trust in the intervention arm, and 5 pregnant women from each participating NHS Trust. We will aim to recruit 7 midwives and 10 pregnant women from each of the relevant pilot sites to allow for withdrawal and loss to follow up.

# Compliance and Withdrawal

Participants have the right to withdraw from the study at any time for any reason, and without giving a reason. It is understood by all concerned that an excessive rate of withdrawals can render the study uninterpretable; therefore, unnecessary withdrawal of patients should be avoided. Should a participant decide to withdraw from the study, all efforts will be made to report the reason for withdrawal as thoroughly as possible.

17.1 Midwives withdrawal

Midwives’ withdrawal from intervention delivery: The risks of the midwives withdrawing (following consent) from the intervention are minimal. The aim is to have all community midwives in the intervention arm receive the intervention, but only 18 randomly selected midwives in each NHS Trust will be participants for outcome data collection (baseline data will have been collected pre-intervention, and follow up data collection to be collected post-intervention). Withdrawal mid-intervention is unlikely as there is only one single day of intervention delivery, so a midwife would have to withdraw part way through the day and leave the training session. Additionally, they would also have to be one of the 18 midwives randomly selected for this to impact on the further outcome data collection. However, it is possible that a midwife may have to leave part way through the intervention delivery (e.g. if they have to leave to collect a sick child from school), or the midwife could not turn up to the intervention (e.g. if they were ill on their allocated intervention day). In both of these circumstances, the midwife will be offered the opportunity to attend another intervention day to complete their training as the intervention will be delivered a number of times in each NHS trust in the intervention arm. If it is not possible for the midwife to re-attend for any reason then we will still aim to collect the follow up data if they are one of the 18 randomly selected midwives for data collection (intention to treat).

Midwives’ withdrawal from data collection: If any of the midwives selected to provide data for the pilot study requests to withdraw then the following actions will be taken:

- Request to completely withdraw from the study - no further data will be collected but we will retain data already provided and use in the analysis (intention to treat).
- Request to withdraw due to change in circumstances (e.g. retire, move to another NHS Trust rather than not wanting to participate further) – as the midwives will no longer be providing care for women in the Trust recruited for the trial, we will retain data already provided and attempt to collect exit data (final outcome data) at the point of withdrawal. Data provided will be used in the analysis (intention to treat).
- Non explicit withdrawal (e.g. non-return of questionnaire), repeat attempts will be made to collect this data (via postal and telephone reminders, up to three total contacts). In the case of non-explicit withdrawal, further attempts will be made to collect data from midwives at future time points.

17.2 Women’s withdrawal

Researcher decision to withdraw women from data collection: Before each attempt at data collection, the local PIs/research midwives will check for adverse pregnancy events (unrelated to the intervention) to avoid contacting women and causing unnecessary distress to them or their families. The type of adverse event that would deem the women to be automatically withdrawn from the study would be the death of the participant, or the death of the baby (including miscarriage, late fetal loss, stillbirth, neonatal death, and infant death up to 1 year postnatal follow up).

Women’s decision to withdraw from data collection:

- Explicitly withdraw from further involvement in the study – no further data will be collected but we will retain data already provided and use in the analysis (intention to treat).
- Non explicit withdrawal (e.g. non-return of questionnaire), repeat attempts will be made to collect this data (via postal and telephone reminders, up to three total contacts). In the case of non-explicit withdrawal, further attempts will be made to collect data from women at future time points.

# Data Monitoring, Quality Control and Quality Assurance

This is a low risk pilot trial and major safety issues are not anticipated. The project advisory group (PAG) includes independent members described in section 1, and will be responsible for monitoring the conduct of the pilot study. The full PAG has had one pre-study meeting in 2015, and will meet again following delivery of the intervention, and annually until the end of the study.

Additional monitoring of study conduct and data collected will be performed by a combination of central review and site monitoring visits to ensure the study is conducted in accordance with GCP. Study site monitoring will be undertaken by NH. Site monitoring will include:

- All original consent forms will be reviewed as part of the study file
- All original consent forms will be compared against the study participant identification list
- The presence of essential documents in the investigator site file and study files will be checked
- Check the procedures and recording for adverse events (unrelated to the study) that would result in automatic withdrawal of women from the study (log of the checks made)
- Check the procedures and recording of explicit withdrawals from the study/data collection, and that mechanisms are in place to stop follow up data collection requests being sent to participants who have explicitly withdrawn

Central monitoring by the CI will include:

- All applications for study authorisations and submissions of progress reports will be reviewed for accuracy and completeness, prior to submission
- All documentation essential for study initiation will be reviewed prior to site authorisation

All monitoring findings will be reported and followed up with the appropriate persons in a timely manner. The study may be subject to inspection and audit by NUTH NHS Trust under their remit as sponsor, and other regulatory bodies to ensure adherence to GCP. The investigators/institutions will permit trial-related monitoring, audits, REC review and regulatory inspections, providing direct access to source data/documents.

# Adverse Event Monitoring and Reporting

The intervention in this pilot study is targeted exclusively at midwives. Therefore any intervention adverse events will not involve pregnant women and adverse events related to the pregnant women will not be captured by the study. However, adverse events which are unrelated to the study will be checked before each data collection contact with pregnant and postnatal women to inform research decisions for withdrawal (as described in section 17.2).

There is low risk of adverse events relating directly to the intervention (midwifery training). There is a minimal possibility that midwives may become upset during the training (e.g. being asked to reflect on their past practice experiences if there has been a particularly negative experience). In this circumstance the following procedures will be put in place:

- Stop the intervention delivery
- Ask the midwife if she would like to leave the room for a while (supported by the observer (NH) who will be present during the intervention delivery)
- Remind the midwife of her option to withdraw from the intervention
- Signpost to follow up staff support in their local NHS trust if necessary (this would involve suggesting that they contact their allocated supervisor of midwives or line manager for further support)
- Any instance of adverse events will be recorded in the study file at the related site.

# Ethics & Regulatory Issues

Favourable ethical opinion from an appropriate REC, and R&D approval, will be sought prior to commencement of the study. Local approvals will be sought before recruitment may commence at each site. The Study Coordination Centre will require a written copy of local approval documentation before initiating each centre and accepting participants into the study. Information sheets will be provided to all eligible participants and written informed consent obtained prior to any study procedures.

# Confidentiality

Personal data will be regarded as strictly confidential. To preserve anonymity, any research data leaving the site will identify participants by a unique participant identification code only. Personal details will be required to organise data collection among consented participants (e.g. to arrange semi-structured interviews). The study will comply with the Data Protection Act, 1998. All study records and investigator site files will be kept at site in a locked filing cabinet with restricted access, and electronic data on a secure network folder, password protected with restricted access.

# Insurance and Finance

The NUTH NHS Trust has liability for clinical negligence that harms individuals toward whom they have a duty of care. NHS Indemnity covers NHS staff and medical academic staff with honorary contracts conducting the trial for potential liability in respect of negligent harm arising from the conduct of the study. The NUTH NHS Trust is Sponsor and through the Sponsor, NHS indemnity is provided in respect of potential liability and negligent harm arising from study management. Indemnity in respect of potential liability arising from negligent harm related to study design is provided by NHS schemes for those protocol authors who have their substantive contracts of employment with the NHS and by Newcastle University Insurance schemes for those protocol authors who have their substantive contract of employment with the University. This is a non-commercial study and there are no arrangements for non-negligent compensation.

The NIHR are funding the study as part of an NIHR Postdoctoral Research Fellowship (NH).

# Study Report / Publications

The data will be the property of the CI and Co-Investigators. Publication will be the responsibility of the CI. It is planned to publish this study in peer review articles and to present data at national and international meetings. Results of the study will also be reported to the Sponsor and Funder, and will be available on their web site. All manuscripts, abstracts or other modes of presentation will be reviewed by the PAG and Funder prior to submission. Individuals will not be identified from any study report.

Participants will be offered to receive information about their contribution to the study at the end of the study, including a lay summary of the results. The results of this study will inform the development of a grant application for a definitive trial.

# References

1. Heslehurst N, Rankin J, Wilkinson JR, et al. A Nationally Representative Study of Maternal Obesity in England, UK: Trends in Incidence and Demographic Inequalities in 619 323 Births, 1989-2007. International Journal of Obesity 2010;**34**:420-28

2. Heslehurst N, Sattar N, Rajasingam D, et al. Existing maternal obesity guidelines may increase inequalities between ethnic groups: a national epidemiological study of 502,474 births in England BMC Pregnancy Childbirth 2012;**12**(156)

3. Torloni MR, Betrán AP, Horta BL, et al. Prepregnancy BMI and the risk of gestational diabetes: a systematic review of the literature with meta-analysis. Obesity Reviews 2008;**10**:194–203

4. Poston L, Harthoon LF, Van der Beek EM, et al. Obesity in Pregnancy: Implications for the Mother and Lifelong Health of the Child. A Consensus Statement. Pediatric Research 2011;**69**(2):175-80

5. Heslehurst N, Simpson H, Ells LJ, et al. The impact of maternal BMI status on pregnancy outcomes with immediate short-term obstetric resource implications: a meta-analysis. Obesity Reviews 2008;**9**(6):635-83

6. Stothard KJ, Tennant PWG, Bell R, et al. Maternal Overweight and Obesity and the Risk of Congenital Anomalies: A Systematic Review and Meta-analysis JAMA 2009;**301**(6):636-50

7. Tennant PWG, Rankin J, Bell R. Maternal body mass index and the risk of fetal and infant death; a cohort study from the North of England. Human Reproduction 2011;**26**(6):1501–11

8. Turcksin R, Bel S, Galjaard S, et al. Maternal obesity and breastfeeding intention, initiation, intensity and duration: a systematic review. Maternal & child nutrition 2014;**10**(2):166-83 doi: 10.1111/j.1740-8709.2012.00439.x

9. Foresight. Tackling Obesities: Future Choices – Project Report: Government Office for Science, Department of Innovation Universities and Skills, 2007.

10. Heslehurst N, Russell S, Brandon H, et al. Women's perspectives are required to inform the development of maternal obesity services: a qualitative study of obese pregnant women's experiences. Health Expectations 2013 doi: 10.1111/hex.12070

11. National Institute for Health and Clinical Excellence. Clinical Guideline 43 - Obesity: Guidance on the prevention of overweight and obesity in adults and children. London: NICE, 2015.

12. National Institute for Health and Clinical Excellence. Public Health Guidance 49: Behaviour change: individual approaches. London: NICE, 2014.

13. Heslehurst N. Identifying groups of 'at risk' women, associated health inequalities and the impact of maternal obesity on NHS maternity services. Proceedings of the Nutrition Society 2011;**70**(4):439-49

14. Alavi N, Haley S, Chow K, et al. Comparison of national gestational weight gain guidelines and energy intake recommendations. Obesity Reviews 2013;**14**(1):68-85 doi: 10.1111/j.1467-789X.2012.01059.x

15. National Institute for Health and Clinical Excellence. Weight Management Before, During and After Pregnancy: Department of Health, 2010

16. Denison F, Norrie G, Graham B, et al. Increased maternal BMI is associated with an increased risk of minor complications during pregnancy with consequent cost implications. BJOG: an International Journal of Obstetrics & Gynaecology 2009;**116**:1467–72

17. Morgan K, Rahman M, Macey S, et al. Obesity in pregnancy: a retrospective prevalence-based study on health service utilisation and costs on the NHS. BMJ Open 2014;**4**:e003983

18. Watson M, Howell S, Johnston T, et al. Pre-pregnancy BMI: Costs associated with maternal underweight and obesity in Queensland. Australian and New Zealand Journal of Obstetrics and Gynaecology 2013;**53**(3):243-49 doi: 10.1111/ajo.12031

19. Heslehurst N, Newham J, Maniatopoulos G, et al. Implementation of pregnancy weight management and obesity guidelines: a meta-synthesis of healthcare professionals' barriers and facilitators using the Theoretical Domains Framework. Obesity Reviews 2014;**15**:462-86 doi: 10.1111/obr.12160

20. Heslehurst N, Moore H, Rankin J, et al. How can maternity services be developed to effectively address maternal obesity? A qualitative study. Midwifery 2011;**27**(5):e170-e77

21. Heslehurst N, Russell S, McCormack S, et al. Midwives perspectives of their training and education requirements in maternal obesity: A qualitative study. Midwifery 2013;**29**(7):736-44 doi: 10.1016/j.midw.2012.07.007

22. Heslehurst N, Lang R, Rankin J, et al. Obesity in pregnancy: a study of the impact of maternal obesity on NHS maternity services. BJOG: an International Journal of Obstetrics & Gynaecology 2007;**114**:334-42

23. Brown I, Thompson J, Tod AM, et al. Primary Care Support for Tackling Obesity: A Qualitative Study of the Perceptions of Obese Patients. British Journal of General Practice 2006;**56**:666-72

24. Merrill E, Grassley J. Women's Stories of their Experiences as Overweight Patients. Journal of Advanced Nursing 2008;**64**(2):139-46

25. Keenan J, Stapleton H. Bonny Babies? Motherhood and Nurturing in the Age of Obesity. Health, Risk & Society 2010;**12**(4):369-83

26. Wiles R. I'm Not Fat, I'm Pregnant. In: Wilkinson S, Kitzinger C, eds. Women and Health: Feminist Perspectives. London: Taylor & Francis, 1994:33-48.

27. Nyman VMK, Prebensen AK, Flensner GEM. Obese women's experiences of encounters with midwives and physicians during pregnancy and childbirth. Midwifery 2010;**26**(4):424-29 doi: [published Online First: Epub Date]|.

28. Heslehurst N, et al. An Evaluation of the Implementation of Maternal Obesity Pathways of Care: a Mixed Methods Study with Data Integration. An Evaluation of the Implementation of Maternal Obesity Pathways of Care: a Mixed Methods Study with Data Integration. PLoS ONE 2015; 10(5): e0127122.

29. CMACE-RCOG. Management of Women with Obesity in Pregnancy: Jointly published by the Centre for Maternal and Child Enquiries and the Royal College of Obstetricians and Gynaecologists, 2010.

30. Michie S, Johnston M, Abraham C, et al. Making psychological theory useful for implementing evidence based practice: a consensus approach. Quality and Safety in Health Care 2005;**14**:26-33

31. Michie S, Pilling S, Garety P, et al. Difficulties Implementing a Mental Health Guideline: An Exploratory Investigation using Psychological Theory. Implementation Science 2007;**2**:8

32. French S, Green S, O'Connor D, et al. Developing theory-informed behaviour change interventions to implement evidence into practice: a systematic approach using the Theoretical Domains Framework. Implementation Science 2012;**7**(1):38

33. Fishbein M. Attitude and the prediction of behavior. Readings in attitude theory and measurement. New York: Wiley, 1967.

34. Bandura A. Health promotion from the perspective of social cognitive theory. Psychology & Health 1998;**13**(4):623-49

35. Nicola Heslehurst, Lisa Crowe, Shannon Robalino, et al. Interventions to change maternity healthcare professionals’ behaviours to promote weight related support for obese pregnant women: a systematic review. Implementation Science 2014;**9**(97)

36. Bandura A. Exercise of human agency through collective efficacy. Current Directions in Psychological Science 2000;**9**(3):75-78

37. Harvey E, Glenny AM, Kirk S, et al. Improving health professionals’ management and the organisation of care for overweight and obese people (Review). Cochrane Database of Systematic Reviews 2009;**1**

38. Flodgren G, Deane K, Dickinson HO, et al. Interventions to change the behaviour of health professionals and the organisation of care to promote weight reduction in overweight and obese adults (Review). Cochrane Database of Systematic Reviews 2010;**12**

39. Flodgren G CL, Mayhew A, Omar O, Pereira CR, Shepperd S. . Interventions to improve professional adherence to guidelines for prevention of device-related infections. Cochrane Database of Systematic Reviews 2013;**Issue 3**(Art. No.: CD006559):DOI: 10.1002/14651858.CD006559.pub2

40. Giguère A LF, Grimshaw J, Turcotte S, Fiander M, Grudniewicz A, Makosso-Kallyth S, Wolf FM, Farmer AP, Gagnon M-P. Printed educational materials: effects on professional practice and healthcare outcomes. Cochrane Database of Systematic Reviews 2012;**Issue 10**(Art. No.: CD004398):DOI: 10.1002/14651858.CD004398.pub3

41. Akl EA KV, Sackett KM, Erdley WS, Mustafa RA, Fiander M, Gabriel C, Schünemann H. Educational games for health professionals. Cochrane Database of Systematic Reviews 2013;**Issue 3**(Art. No.: CD006411):DOI: 10.1002/14651858.CD006411.pub4.

42. Baker R C-SJ, Gillies C, Shaw EJ, Cheater F, Flottorp S, Robertson N, Wensing M, Fiander M, Eccles MP, Godycki-Cwirko M, van Lieshout J, Jäger C. Tailored interventions to address determinants of practice. Cochrane Database of Systematic Reviews 2015;**Issue 4**(Art. No.: CD005470):DOI: 10.1002/14651858.CD005470.pub3

43. Forsetlund L BA, Rashidian A, Jamtvedt G, O'Brien MA, Wolf FM, Davis D, Odgaard-Jensen J, Oxman AD. Continuing education meetings and workshops: effects on professional practice and health care outcomes. Cochrane Database of Systematic Reviews 2009; **Issue 2**(Art. No.: CD003030):DOI: 10.1002/14651858.CD003030.pub2

44. O'Brien MA RS, Jamtvedt G, Oxman AD, Odgaard-Jensen J, Kristoffersen DT, Forsetlund L, Bainbridge D, Freemantle N, Davis D, Haynes RB, Harvey E. Educational outreach visits: effects on professional practice and health care outcomes. Cochrane Database of Systematic Reviews 2007;**Issue 4**(Art. No.: CD000409):DOI: 10.1002/14651858.CD000409.pub2

45. Thomas LH CN, McColl E, Rousseau N, Soutter J, SteenN. Guidelines in professions allied to medicine. Cochrane Database of Systematic Reviews 1999;**Issue 1**(Art. No.: CD000349):DOI: 10.1002/14651858.CD000349

46. Michie S, Ashford S, Sniehotta FF, et al. A refined taxonomy of behaviour change techniques to help people change their physical activity and healthy eating behaviours: the CALO-RE taxonomy. Psychol Health 2011;**26**(11):1479-98 doi: 10.1080/08870446.2010.540664[published Online First: Epub Date]|.

47. Michie S, Johnston M, Francis J, et al. From Theory to Intervention: Mapping Theoretically Derived Behavioural Determinants to Behaviour Change Techniques. Applied Psychology 2008;**57**(4):660-80 doi: 10.1111/j.1464-0597.2008.00341.x[published Online First: Epub Date]|.

48. Medical Research Council. Developing and evaluating complex interventions: new guidance, 2008.

49. Lancaster GA, Dodd S, Williamson PR. Design and Analysis of Pilot Studies: Recommendations for Good Practice. Journal of Evaluation in Clinical Practice 2004;**10**(2):307-12

50. Thabane L, Ma J, Chu R, et al. A tutorial on pilot studies: the what, why and how. BMC Medical Research Methodology 2010;**10**(1) doi: doi:10.1186/1471-2288-10-1[published Online First: Epub Date]|.

51. Campbell MK, Miollison J, Grimshaw JM. Cluster Trials in Implementation Research:Estimation of Intracluster Correlation Coefficients and Sample Size. Statistics in Medicine 2001;**20**:391-99

52. Hoffmann TC, Glasziou PP, Boutron I, et al. *Better reporting of interventions: template for intervention description and replication (TIDieR) checklist and guide*, 2014.

# Appendices

Appendix 1: Social Cognitive Theory Model for Weight Communication Behaviours

Appendix 2: Social Cognitive Theory Model for Weight Management Behaviours

Appendix 3: Hypothesised pathway to midwives and women’s behaviour change, and associated populations and time points for economic evaluation outcome measures

Appendix 4: Anticipated cost and outcome information relevant to a definitive trial and economic modelling

Appendix 5: Hypothesised balance sheet of the costs and benefits relevant to a cost-effectiveness analysis

Appendix 6: Overview of the Intervention Day

Appendix 7: Graded Tasks for Weight Communication Intervention Component

Appendix 8: Graded Tasks for Weight Management Intervention Component

Appendix 9: Graded Tasks for Consolidation Intervention Component

Appendix 1: Social Cognitive Theory Model for Weight Communication Behaviours (Note: bold font indicates evidence of being a strong barrier to practice)

Appendix 2: Social Cognitive Theory Model for Weight Management Behaviours (Note: bold font indicates strong evidence of being a barrier to practice)

Appendix 2 continued


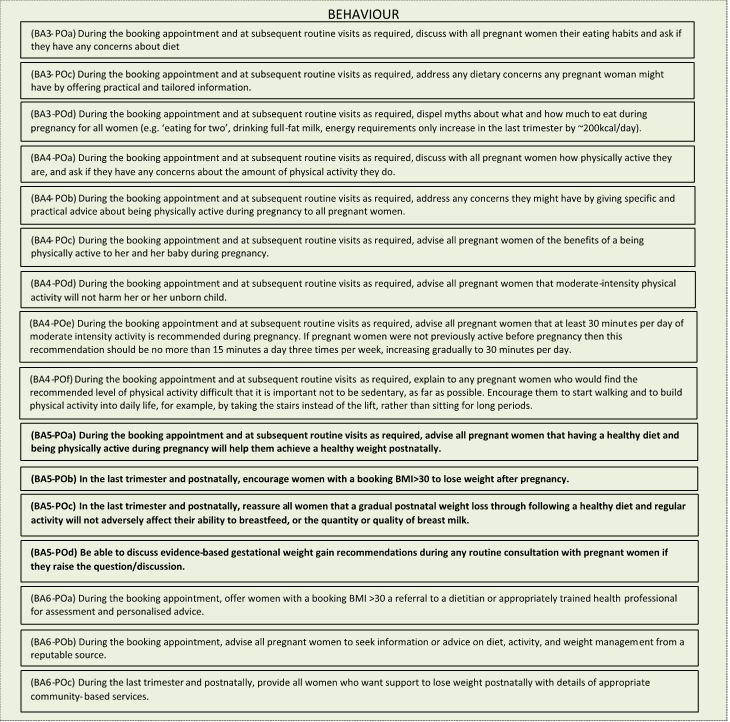


Appendix 3: Hypothesised pathway to midwives and women’s behaviour change, and associated populations and time points for economic evaluation outcome measures

Intervention delivery

- One day intensive training for community midwives

Primary aim/outcome: Midwives behaviour change (midwives adherence to guideline recommendations for health professionals’ provision of weight management advice and support)

- 1 month post intervention
- 6 months post intervention

Secondary aims/outcomes: Midwives behaviour change mediates women’s behaviour change (women’s adherence to guideline recommendations for women’s diet and PA behaviours)

- Baseline (asap after booking)
- 3^rd^ trimester
- Postnatal (3, 6, 9, 12 months)
- Health outcomes for the woman
  - Short term
    - Pregnancy
    - Postnatal (3, 6, 9, 12 months)
  - Long term
    - Modelled outcomes
- Health outcomes for the child
  - Short term
    - Pregnancy
    - Postnatal (6, 12 months)
  - Long term
    - Modelled outcomes

Appendix 4: Anticipated cost and outcome information relevant to a definitive trial and economic modelling

| **Measure** | **Collection method** | **Timing of collection** |
| --- | --- | --- |
| **Intervention** |  |  |
| Training pack unit production costs (1 per midwife) | Trial budget records, purchase orders, invoices | During production |
| Pregnant women information pack unit production cost (1 per pregnant woman) | Trial budget records, purchase orders, invoices | During production |
| Number of midwives invited to each training session | Trial training records | Throughout training |
| Number of attending midwives at each training session | Consent forms | End of trial |
| One day of midwife time out of practice to attend training session | Information from participating pilot sites about the salary band for their community midwives | Pre-trial |
| Midwife preparation time for training day | Evaluation form | On day of training |
| Further optional midwife CPD as a direct result of attending the training (e.g. midwife time spent doing further reading, using online resources, and which sites used etc) | Midwife self-report questionnaire | 3 months post training |
| Midwife travel costs to attend training | Expense claim form | Throughout training |
| Training facilitator time cost (on day and prep time) | Facilitator time log, salary details from trial budget records | Throughout training |
| Costs of refreshments and thank you packs for midwives attending the training sessions | Trial budget records, purchase orders, invoices | Throughout pilot study |
| Admin support time to assist with preparation of training days (e.g. ordering refreshments, printing etc) | Time log | Throughout pilot study |
| Number of training sessions | Time log | Throughout pilot study |
| Room hire costs | Trial budget records, purchase orders, invoices | Throughout pilot study |

| **Measure** | **Collection method** | **Timing of collection** |
| --- | --- | --- |
| **Midwife behaviour** | |  |
| Midwife baseline characteristics (number of years in practice as a midwife/as a community midwife, past training on maternal obesity or weight mangement, educational attainment, age range, BMI range?) | Questionnaire | Pre-intervetion |
| Adherence to guidelines | Midwife self-report questionnaire | Baseline, 1 month after training, 6 months after training |
|  | Pregnant women self-report questionnaire | After all midwives have received training (approx 6 months after starting to deliver the training), collected from women as soon as possible after their booking appointment |
|  | Case note audit | After discharge |
|  | Midwives simulated practice (vignettes) | Baseline, 1 month after training, 6 months after training |
| Social-cognitive theory constructs of the behaviour (self-efficacy, outcome expectancies, goals, knowledge, attitudes) | Questionnaires | Baseline, a month after training, 6 months after training |
| Time spent discussing maternal weight and weight management in practice | Simulated practice (timed role play during training) | During training |
|  | Midwife self-report questionnaire | Baseline, 1 month after training, 6 months after training |
| Prep time for booking appointment consultations and follow-up time post booking appointment relating to weight management (e.g. time spent finding information to answer pregnant womens questions on weight management if they can't answer during the consultation) | Midwife self-report questionnaire | Baseline, 1 month after training, 6 months after training |
| Identification of additional important information relating to midwives adherence to the guidelines not captured with above measures (e.g. detailed account of midwives successes/difficulties in adhering to the guidelines, behavioural constructs, issues with intervention delivery or translation to practice) | Focus groups with midwives | 1 month after training, 6 months after training |

| **Measure** | **Collection method** | **Timing of collection** |
| --- | --- | --- |
| **Women's behaviour** |  |  |
| Baseline characteristics at booking (BMI, parity, smoking status, age, ethnic group, post code [to determine socio-economic status using Index of Multiple Deprivation], employment, qualifications, stage of pregnancy at booking, past caesarean section, history of diabetes or gestational diabetes, alcohol consumption, maternal medical history [history of depression, thromboembolism, pre-existing essential hypertension]) | Electronic hospital records and maternity case notes (recorded at booking appointment) | After recruitment and consent - post-booking |
| Dietary and physical activity patterns | Women self-report validated questionnaires | Baseline, 3rd trimester, post-natal (3,6,9,12 months) |
| Engagement with referrals/support services for weight management | Women self-report questionnaires, attendance rates at existing weight management clinics (e.g. dietetics service etc) from hospital appointment system | Baseline, 3rd trimester, post-natal (3,6,9,12 months) |
| Costs incurred by women managing weight (gym membership, equipment purchased, attending ante-natal classes, family food costs, child care) | Qualitative interview | 3rd trimester, 6, 12 months post-natal |
| Additional appointments with community midwife relating specifically to weight management | Case note audit | After discharge |

| **Measure** | **Collection method** | **Timing of collection** |
| --- | --- | --- |
| **Women's short term health outcomes** |  |  |
| Gestational weight gain | Direct measurement | 3rd trimester |
| Weight retention | Direct measurement | 3,6,9,12 months post-natal |
| Psychosocial well-being measure | Self-report questionnaire | Baseline, 3rd trimester, post-natal (3,6,9,12 months) |
| Mode of delivery (spontaneous, induced, assisted vaginal, unassisted vaginal, elective caesarean, emergency caesarean, analgesia use) | Electronic patient records; case notes | After discharge |
| Health service usage costs (outpatient appointments, maternity admissions, length of postnatal stay) | Electronic patient records; case notes | After discharge |
| Pregnancy and delivery complications (preeclampsia, gestational diabetes, gestational hypertension, postpartum haemorrhage, infection, VTE in pregnancy) | Electronic patient records; case notes | After discharge |

| **Measure** | **Collection method** | **Timing of collection** |
| --- | --- | --- |
| **Women's longer term health outcomes** |  |  |
| Future risk of type 2 diabetes | To be modelled | NA |
| Long-term weight status | To be modelled | NA |
| Long term breastfeeding health benefits | To be modelled | NA |
| Future pregnancy burden (gestational diabetes, caesarean sections, etc.) | To be modelled | NA |

| **Measure** | **Collection method** | **Timing of collection** |
| --- | --- | --- |
| **Child's short term health outcomes** |  |  |
| Birthweight (birth weight, plus sex and gestational age to calculate SGA and LGA percentiles) | Mother's case notes, electronic patient record | After discharge |
| Intrauterine death | Mother's case notes, electronic patient record | After discharge |
| Complications (shoulder dystocia, birth trauma, respiratory distress, infant hypoglycaemia, apgar score 1 and 5 mins, infant hyperbilirubinaemia) | Mother's case notes, electronic patient record | After discharge |
| Admitted to NICU/SCBU (reason and duration) | Baby's case notes | After discharge |
| Child growth | Childs red book health record if recorded, direct measure by research midwife if not | 6, 12 months postnatal |
| Breastfeeding (exclusive, duration, age at cessation, age at weaning) | Patient records for breastfeeding status at discharge | After discharge |
|  | Questionnaire continued breastfeeding and weaning | 3,6,9,12 months postnatal |

| **Measure** | **Collection method** | **Timing of collection** |
| --- | --- | --- |
| **Child's longer term health outcomes** |  |  |
| Long term weight status | To be modelled | NA |
| Future risk of type 2 diabetes | To be modelled | NA |
| Long term breastfeeding health benefits | To be modelled | NA |

Appendix 5: Hypothesised balance sheet of the costs and benefits relevant to a cost-effectiveness analysis

| **Costs** | **Benefits** |
| --- | --- |
| **Intervention** |  |
| Training pack production |  |
| Women information pack production |  |
| Training delivery |  |
| Midwife training time and travel |  |
| **Midwife behaviour** |  |
| Midwife time advising pregnant women | Midwife work-related stress |
| **Pregnant women behaviour** |  |
| Pregnant women diet and exercise expense |  |
| Use of referrals and support services |  |
| Appointments with community midwives |  |
| **Pregnant women short term outcomes** |  |
| Pregnancy and delivery complications | Pregnancy and delivery complications |
| Delivery costs | Psychosocial wellbeing measure |
| Other health service use |  |
| **Pregnant women long term outcomes** |  |
| Future risk of type 2 diabetes related resource use | Future risk of type 2 diabetes |
| Long-term weight health effects related resource use | Long-term weight-related health outcomes |
| Long-term breastfeeding health effects related resource use | Long-term breastfeeding health benefits |
| Future pregnancy burden (gestational diabetes, caesarean sections, etc.) related resource use | Future pregnancy health complications (gestational diabetes, caesarean sections, etc.) |
| **Baby short term outcomes** |  |
| Baby complications at birth related resource use | Baby complications at birth |
| Admission to NICU/SCBU | Intrauterine death |
| Long-term low birthweight related health effect resource use | Long-term low birthweight related health outcomes |
| **Baby long term outcomes** |  |
| Long-term weight health effects related resource use | Long-term weight-related health outcomes |
| Future risk of type 2 diabetes related resource use | Future risk of type 2 diabetes |
| Long-term breastfeeding health effects related resource use | Long-term breastfeeding health benefits |

Appendix 6: Overview of the Intervention Day

08.30-08.50 Session 1: Introduction (with hot drinks)

08.50-10.30 Session 2: Weight Communication

*Coffee break – refreshments provided*

10.45-12.00 Session 2 continued: Weight Communication

*Lunch break – lunch provided*

12.45-14.45  Session 3: Weight management

*Coffee break – refreshments provided*

15.00-16.00 Session 4: Consolidation of the day

16.00-16.30 Session 5: Summary and evaluation forms

Appendix 7: Graded Tasks for Weight Communication Intervention Component

| **Graded Task** | **Session 2: Weight Communication** |
| --- | --- |
| 1. **Provide evidence-base/ guidelines** | **Quiz/interactive game to allow midwives to demonstrate their existing knowledge of obesity communication and risks; research evidence and guidelines prepared as a lecture**   - Maternal obesity definitions and risks to the mother and baby - Obesity determinants, stigma (society and healthcare settings), and guidance for sensitive communication - Women's perspectives of weight-related communication, terminology, negative and positive reactions to weight-related communication (causes of reaction types) – with audio - Maternal obesity risks (mother and baby) and risk management   **Provision of educational materials incorporated into a training pack for midwives**   - Copies of slides with space for notes, references used in the lecture, key papers used as evidence base - Further reading/resources on this topic (e.g. additional reference list/websites etc) |
| 1. **Demonstrate** | **Video with audio**   - A midwife raising the topic of weight and risk management with an obese pregnant woman - Some elements of verbal communication that are sensitive and that could be improved   **Muted video**   - A muted consultation between a midwife and obese woman - some elements of non-verbal communication that are sensitive/non-stigmatising and that could be improved   **Group discussion of video demonstration to include:**   - Awareness/identification of sensitivity, stigma and constructive risk communication |
| 1. **Reflect on own practice** | **Individual reflection exercise**:   - Experience of positive/negative weight-related and risk communication in practice - Own weight status and impact on attitudes and practice - Their own/other peoples obesity stereotypes and prejudices in the workplace   **Group discussion of individual reflection to include:**   - Shared experiences, extreme experiences (positive and negative, frequency), “every day” experiences (positive and negative and neutral) |
| 1. **Provide instruction** | **Script - midwives contribute to developing throughout the day**   - Script content - raising the topic of weight in pregnancy using sensitive communication, providing constructive risk and risk management communication   **Provision of new and existing resources for practice**   - Resources to facilitate sensitive weight and risk communication (e.g. to use in practice to break down barriers to initiating the discussion, pathways of care)   **Provision of educational materials incorporated into a training pack for midwives**   - Hard copies of educational materials incorporated into a training pack |
| 1. **Practice** | **Role-play**   - Groups of 3 (one role playing the midwifery role, one the pregnant woman role, one the observer) - each gets the opportunity to practice each role with positive/negative responses |
| 1. **Feedback** | **Feedback from facilitator**   - Praise for effort and success   **Group discussion/feedback from peers**   - How they felt before, during and after doing the role play; how their partners performed as a midwife and as a pregnant woman (positive and areas for improvement) |
| 1. **Reflection on performance** | **Individual reflection exercise**   - How they feel now about raising the topic of weight in pregnancy - How they feel about verbal and non-verbal communication - How they feel now about communicating constructive obesity risks and risk management   **Adaptation of the script to reflect their own wording**   - Based on positive reflections from their clinical practice and this training |

Appendix 8: Graded Tasks for Weight Management Intervention Component

| **Graded Task** | **Session 3 – Weight management** |
| --- | --- |
| 1. **Provide evidence-base/ guidelines** | **Quiz/interactive game to allow midwives to demonstrate their existing knowledge of weight management; research evidence and guidelines prepared as a lecture**   - Published guidelines relating to weight management, including dietary and physical activity recommendations, myths and safety - Published evidence-base on the effectiveness/benefits of weight management interventions to improve health/reduce risk to the mother and fetus/baby/child - Published evidence base on women’s motivation and engagement with weight management behaviours during pregnancy - Guidelines/evidence-base relating to impact of breastfeeding on weight management, and vice versa - Availability of local support for weight management for pregnant and postnatal women   **Provision of educational materials incorporated into a training pack for midwives**   - Copies of slides with space for notes, references used in the lecture, key papers used as evidence base - Further reading/resources on this topic (e.g. additional reference list/websites etc) |
| 1. **Demonstrate** | **Video with audio**   - A midwife discussing weight management with an obese pregnant woman including: health benefits, dietary/PA requirements, myths, sharing information resources, providing tailored and practical information/responding to cues from the woman, introducing postnatal weight loss/breastfeeding   **Group discussion of video demonstration to include:**   - Awareness and identification of sensitive/insensitive weight management information, guideline recommendations, opportunities for a midwife to tailor the information provided to women’s needs, and provide practical advice |
| 1. **Reflect on own practice** | **Individual reflection exercise**:   - Experience of positive/negative weight management practice and women’s motivation   **Group discussion:**   - Shared experiences, extreme experiences (positive and negative, frequency), “every day” experiences (positive and negative and neutral) |
| 1. **Provide instruction** | **Script - midwives contribute to developing throughout the day**   - Script content - practical, tailored, benefits, safety of weight management in pregnancy   **Provision of new and existing resources for practice**   - Resources to facilitate weight management discussion (e.g. information for women)   **Provision of educational materials incorporated into a training pack for midwives**   - Hard copies of educational materials incorporated into a training pack |
| 1. **Practice** | **Role-play**   - Groups of 3 (one role playing the midwifery role, one the pregnant woman role, one the observer) - each gets the opportunity to practice each role with positive/negative responses |
| 1. **Feedback** | **Feedback from facilitator**   - Praise for effort and success   **Group discussion/feedback from peers**   - How they felt before, during and after doing the role play; how their partners performed as a midwife and as a pregnant woman (positive and areas for improvement) |
| 1. **Reflection on performance** | **Individual reflection exercise**   - How they feel about discussing weight management, providing practical/tailored advice   **Adaptation of the script to reflect their own wording**   - Based on positive reflections from their clinical practice and this training |

Appendix 9: Graded Tasks for Consolidation Intervention Component

| **Tasks** | **Session 4 – consolidation session** |
| --- | --- |
| **1. Practice** | **Timed role play**   - Consolidation of all previous role plays, timed role-play to identify time required to perform the behaviour in practice |
| **2. Prioritising** | **Reflection**  • Reflect on practice and identify competing priorities and when weight management should be prioritised  • Prioritise and plan which aspects of weight management to deliver under time pressures and other competing priorities |
| **3. Action planning** | **Identify what they should change, can change, and how to change**   - Identify which aspects of the training they think they should implement into practice? - Identify which aspects of the training will be easy to implement into practice, which aspects will be more difficult? - Plans on how to implement changes to practice (e.g. how to incorporate awareness of verbal and non-verbal stigma into their communication) |
| **4. Coping planning** | **Develop if/then plans**  Based on their personally identified difficulties/prioritising and responding to cues from the woman, e.g:  **If** I notice that I am judging the women as soon as I see her, **then** I will remind myself of the stigma that they experience to be more compassionate  **If** a woman has a high BMI and smokes, **then** I will prioritise smoking and discuss …[x,y,z]... aspects of weight management  **If** a woman has a high BMI and smokes but doesn’t engage with smoking discussions, **then** I will prioritise weight management discussions  **If** a woman becomes upset by the discussion of weight**, then** I will explain the risk management as a minimum, and leave the provide weight management patient information with her to be discussed at a later stage if she wants to |
