# Supplementary material for: Midwives’ survey of their weight management practice before and after the GLOWING guideline implementation intervention: A pilot cluster randomised controlled trial
Source: PLoS One. 2023 Jan 20;18(1):e0280624. doi: 10.1371/journal.pone.0280624 (PMC9858407; doi:10.1371/journal.pone.0280624)
Supplement: S1 Fig — Note, Behaviour categories and sub-categories are: 1. Communication-related behaviours (sub-categories: weight communication and risk communication) 2. Support and intervention-related behaviours (sub-categories diet and nutrition, physical activity, weight management, referrals and signposting). (DOCX) [file pone.0280624.s002.docx]

**S1 Fig: Flowchart of NICE Guideline behaviours relevant to midwifery practice grouped into thematic behaviour categories and sub-categories for communication-related behaviours and behaviour support/intervention-related behaviours.**


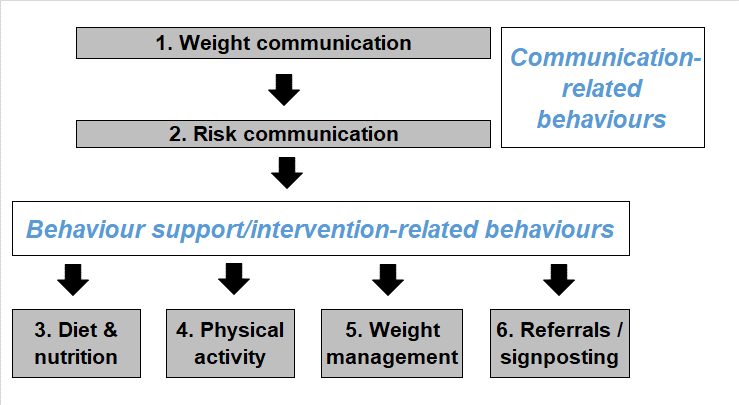


Note, Behaviour categories and sub-categories are:

1. Communication-related behaviours (sub-categories: weight communication and risk communication)

2. Support and intervention-related behaviours (sub-categories diet and nutrition, physical activity, weight management, referrals and signposting)
