# Supplementary material for: Midwives’ survey of their weight management practice before and after the GLOWING guideline implementation intervention: A pilot cluster randomised controlled trial
Source: PLoS One. 2023 Jan 20;18(1):e0280624. doi: 10.1371/journal.pone.0280624 (PMC9858407; doi:10.1371/journal.pone.0280624)
Supplement: S2 Table — (DOCX) [file pone.0280624.s004.docx]

**S3 Table: Adapted NICE guideline recommended behaviours developed for the GLOWING questionnaire**

| **Behaviour Category** | **Adapted recommendation** |
| --- | --- |
| **Weight Communication** | 1. During the booking appointment, explain to all pregnant women why BMI is required 2. During the booking appointment, explain to all pregnant women how BMI will be used to plan their subsequent care 3. Discuss weight status (their individual BMI) with all pregnant women 4. Use sensitive language when discussing weight with pregnant women who have a BMI in the obese range 5. Address the weight-related concerns of pregnant women who have a BMI in the obese range (e.g. worries they have about weight gain) |
| **Risk Communication** | 1. During the booking appointment, explain the pregnancy-related risks to themselves when women have a BMI in the obese range 2. During the booking appointment, explain the risks to their baby to women with a BMI in the obese range 3. During the booking appointment, explain to women with an obese BMI that they should not try to lose weight while pregnant 4. During the booking appointment, explain how the obesity risks will be managed by health professionals caring for them during pregnancy 5. During the booking appointment, explain to women with an obese BMI the risks of gaining too much weight during pregnancy |
| **Diet and Nutrition** | 1. Discuss eating habits with all pregnant women 2. Ask all pregnant women if they have any concerns about their diet 3. Give all pregnant women practical and tailored advice about their diet 4. Discuss pregnancy myths about what and how much to eat during pregnancy with all pregnant women |
| **Physical Activity** | 1. Ask all pregnant women about their levels of physical activity 2. Ask all pregnant women if they have any concerns about the amount of physical activity they do 3. Explain to all pregnant women the benefits of being physically active for their own health 4. Explain to all pregnant women the benefits of being physically active for their baby 5. Advise all women that moderate-intensity physical activity is safe to her and her baby 6. Advise all pregnant women of the number of minutes per day they should be active 7. Explain to all pregnant women that they should avoid being sedentary as much as possible 8. Provide practical advice for all pregnant women about how to build physical activity into their daily life |
| **Weight Management** | 1. Advise all pregnant women that having a healthy diet and being active during pregnancy will help them to achieve a healthy weight postnatally 2. In the last trimester, encourage pregnant women who had a booking BMI in the obese range to lose weight after their pregnancy 3. Encourage women with an obese booking BMI to lose weight when you see them postnatally 4. Reassure women that a gradual postnatal weight loss will not adversely affect their ability to breastfeed 5. Reassure women that a gradual postnatal weight loss will not adversely affect their quantity of breast milk 6. Reassure women that a gradual postnatal weight loss will not adversely affect their quality of breast milk 7. Discuss evidence-based gestational weight gain recommendations with women when they ask you about weight gain in pregnancy |
| **Referrals and Signposting** | 1. Refer all women with an obese BMI to a dietitian or appropriately trained health professional for assessment and personalised advice 2. Advise all pregnant women to seek information or advice on diet, activity, and weight management from a reputable source 3. Provide details of appropriate community-based services to all women who want support to lose weight postnatally |
