# Supplementary material for: Midwives’ survey of their weight management practice before and after the GLOWING guideline implementation intervention: A pilot cluster randomised controlled trial
Source: PLoS One. 2023 Jan 20;18(1):e0280624. doi: 10.1371/journal.pone.0280624 (PMC9858407; doi:10.1371/journal.pone.0280624)
Supplement: S3 Table — * Questions required reverse coding. (DOCX) [file pone.0280624.s005.docx]

**S4 Table: Sum score behaviour categories derived from the questionnaire items for each SCT construct**

| **SCT construct** | **Behaviour category** | **Behaviour sub-category** | **Questionnaire items** |
| --- | --- | --- | --- |
| **Self-efficacy** | Communication-related behaviours | Weight communication | I am confident in my weight-related communication skills when discussing weight status with obese women.  I am confident that I have the skills to sensitively verbally communicate when discussing weight status with obese women.  I am confident that my non-verbal communication skills are sensitive when discussing weight status with obese women. |
|  |  | Risk communication | I am confident in my knowledge of risks to the mother when discussing risks of having a BMI in the obese range.  I am confident in my knowledge of risks to the baby when discussing risks of having a BMI in the obese range.  I am confident that my verbal risk communication skills are sensitive to women with a BMI in the obese range.  I am confident that my non-verbal risk communication skills are sensitive to women with a BMI in the obese range. |
|  | Support- and intervention-related behaviours | Diet, nutrition, physical activity | I am confident in my ability to provide practical and tailored dietary advice to pregnant women.  I am confident in my ability to provide practical and tailored physical activity advice to pregnant women.  I am confident in my knowledge of the benefits of physical activity during pregnancy.  I am confident in my knowledge of the safety of physical activity in pregnancy. |
|  |  | Weight management | I am confident my ability to provide evidence based weight management advice to pregnant women.  I am confident in my ability to provide evidence based weight management advice to postnatal women.  I am confident in my knowledge of evidence based weight management strategies.  I am confident in my knowledge of the impact of postnatal weight loss on women’s ability to breastfeed.  I am confident in my knowledge of the impact of postnatal weight loss on the quality of breast milk.  I am confident in my knowledge of the gestational weight gain evidence base.  I am confident in my ability to advise women about evidence based gestational weight gain recommendations. |
|  |  | Referrals and signposting | I am confident in my knowledge of existing weight management support services available for pregnant women.  I am confident in my knowledge of existing weight management support services available for postnatal women.  I am confident in my knowledge of reputable sources of weight management information for pregnant women. |
| **Outcome expectancies** (negative outcome expectancies reverse coded*, higher score = more positive outcome expectancy) | Communication-related behaviours | Weight communication | If I discuss weight status with pregnant women who have a BMI in the obese range then they will react negatively.*  If I discuss weight status with pregnant women who have a BMI in the obese range then they will become upset anxious angry (etc).*  If I discuss weight status with pregnant women who have obese BMI then it will negatively impact on the relationship I have with them.*  If I discuss weight status with pregnant women who have obese BMI the it will have a negative impact on my emotions (e.g. I will feel upset about the discussion, or worried about them, or fearful of being reported).* |
|  |  | Risk communication | If I discuss obesity risks with pregnant women then they will react negatively.*  If I discuss obesity risks with pregnant women then they will become upset anxious angry (etc).*  If I discuss obesity risks with pregnant women then it will cause harm to the woman.*  If I discuss obesity risks with pregnant women then they will try to lose weight to reduce risks.*  If I discuss risks of gaining too much weight with pregnant women who have an obese BMI then they will react negatively.*  If I discuss risks of gaining too much weight with pregnant women who have an obese BMI then they will try to lose weight.* |
|  | Support- and intervention-related behaviours | Diet, nutrition, physical activity | If I support pregnant women with their weight management then it will help to have a healthier diet.  If I support pregnant women with their weight management then it will help them to be more physically active. |
|  |  | Weight management | If I support pregnant women with their weight management then it will help to minimise gestational weight gain.  If I support pregnant women with their weight management then it will help to reduce risks to the mother.  If I support pregnant women with their weight management then it will help to reduce risk to the baby.  If I support pregnant women with their weight management then it will help to minimise their postnatal weight retention.  If I support pregnant women with their weight management then it will help them to lose weight postnatally. |
|  |  | Referrals and signposting | If I refer pregnant women to an antenatal weight management service they will not want to attend.*  If I refer women for postnatal weight management support they will not want to attend.* |
| **Intentions** | Communication-related behaviours | Weight communication | I intend to explain to all pregnant women why BMI is required during the booking appointment.  I intend to explain to all pregnant women how BMI will be used to plan their subsequent care during the booking appointment.  I intend to discuss weight status (women’s individual BMI) with all pregnant women  I intend to use sensitive language when discussing weight with pregnant women with a BMI in the obese range.  I intend to address the weight-related concerns of pregnant women with a BMI in the obese range (e.g. worries they have about weight gain). |
|  |  | Risk communication | I intend to explain to women the obesity-related risks to them.  I intend to explain to women the obesity related risks to their baby.  I intend to explain to women with a BMI in the obese range that they should not try to lose weight while pregnant.  I intend to explain to women how the obesity risks will be managed by the health professionals caring for them during pregnancy.  I intend to explain to women with an obese BMI the risks of gaining too much weight during pregnancy. |
|  | Support- and intervention-related behaviours | Diet and nutrition | I intend to discuss eating habits with all pregnant women.  I intend to ask all pregnant women if they have any concerns about their diet.  I intend to give all pregnant women practical and tailored advice about their diet.  I intend to discuss pregnancy myths about what and how much to eat during pregnancy with all pregnant women. |
|  |  | Physical activity | I intend to ask all pregnant women about their levels of physical activity.  I intend to ask all pregnant women if they have any concerns about the amount of physical activity they do.  I intend to explain to all pregnant woman the benefits of being physically active for their own health.  I intend to explain to all pregnant women the benefits of being physically active for their baby.  I intend to advise all women that moderate intensity activity is safe to her and her baby.  I intend to advise all pregnant women of the number of minutes per day they should be active.  I intend to explain to all pregnant women that they should avoid being sedentary as much as possible.  I intend to provide practical advice for all pregnant women about how to build physical activity into their daily life. |
|  |  | Weight management | I intend to advise all pregnant woman that having a healthy diet and being active during pregnancy will help them to achieve a healthy weight postnatally.  I intend to encourage pregnant women with an obese booking BMI to lose weight after pregnancy when I see them in the last trimester.  I intend to encourage women with a booking BMI in the obese range to lose weight when I see them postnatally.  I intend to reassure women that a gradual postnatal weight loss through following a healthy diet and regular activity will not adversely affect their ability to breastfeed.  I intend to reassure women that a gradual postnatal weight loss through following a healthy diet and regular activity will not adversely affect the quality of breast milk.  I intend to reassure women that a gradual postnatal weight loss through following a healthy diet and regular activity will not adversely affect the quantity of breast milk.  I intend to discuss evidence based gestational weight gain recommendations with women if they ask me about weight gain. |
|  |  | Referrals and signposting | I intend to refer women with a booking BMI in the obese range a referral to a dietitian or appropriately trained health professional for assessment and personalised advice.  I intend to advise all pregnant women to seek information or advice on diet activity and weight management from a reputable source.  I intend to provide details of appropriate community based services to all women who want support to lose weight postnatally. |
| **Behaviour** | Communication-related behaviours | Weight communication | I explain to all pregnant women why BMI is required.  I explain to all pregnant women how BMI will be used to plan their subsequent care.  I discuss weight status (their individual BMI) with all pregnant women.  I use sensitive language when discussing weight with pregnant women who have a BMI in the obese range.  I address the weight-related concerns of pregnant women who have a BMI in the obese range (e.g. worries they have about weight gain). |
|  |  | Risk communication | I explain the pregnancy-related risks to themselves when women have a BMI in the obese range.  I explain the risks to their baby to women with a BMI in the obese range.  I explain to women with an obese BMI that they should not try to lose weight while pregnant.  I explain how the obesity risks will be managed by health professionals caring for them during pregnancy.  I explain to women with an obese BMI the risks of gaining too much weight during pregnancy. |
|  | Support- and intervention-related behaviours | Diet and nutrition | I discuss eating habits with all pregnant women.  I ask all pregnant women if they have any concerns about their diet.  I give all pregnant women practical and tailored advice about their diet.  I discuss pregnancy myths about what and how much to eat during pregnancy with all pregnant women. |
|  |  | Physical activity | I ask all pregnant women about their levels of physical activity.  I ask all pregnant women if they have any concerns about the amount of physical activity they do.  I explain to all pregnant women the benefits of being physically active for their own health.  I explain to all pregnant women the benefits of being physically active for their baby.  I advise all women that moderate intensity physical activity is safe to her and her baby.  I advise all pregnant women of the number of minutes per day they should be active.  I explain to all pregnant woman that they should avoid being sedentary as much as possible.  I provide practical advice for all pregnant women about how to build physical activity into their daily life. |
|  |  | Weight management | I advise all pregnant women that having a healthy diet and being active during pregnancy will help them to achieve a healthy weight postnatally.  In the last trimester, I encourage pregnant women who had a booking BMI in the obese range to lose weight after their pregnancy.  I encourage women with an obese booking BMI to lose weight when I see them postnatally.  I reassure women that a gradual postnatal weight loss will not adversely affect their ability to breastfeed.  I reassure women that a gradual postnatal weight loss will not adversely affect their quantity of breast milk.  I reassure women that a gradual postnatal weight loss will not adversely affect their quality of breast milk.  I discuss evidence-based gestational weight gain recommendations with women when they ask me about weight gain in pregnancy. |
|  |  | Referrals and signposting | I refer all women with an obese BMI to a dietitian or appropriately trained health professional for assessment and personalised advice.  I advise all pregnant women to seek information or advice on diet activity and weight management from a reputable source.  I provide details of appropriate community based services to all women who want support to lose weight postnatally. |

* Questions required reverse coding
