# Supplementary material for: Midwives’ survey of their weight management practice before and after the GLOWING guideline implementation intervention: A pilot cluster randomised controlled trial
Source: PLoS One. 2023 Jan 20;18(1):e0280624. doi: 10.1371/journal.pone.0280624 (PMC9858407; doi:10.1371/journal.pone.0280624)
Supplement: S4 Table — *Note, 21 midwives only returned one questionnaire, two of these only returned their post-intervention questionnaire therefore have missing pre-intervention data. (DOCX) [file pone.0280624.s006.docx]

**S5: Midwife characteristics and scores for the behaviour categories and Social Cognitive Theory (SCT) constructs, comparing those who returned one or both questionnaires**

|  | Only one questionnaire returned (n=19*) | | Both questionnaires returned (n=47) | |
| --- | --- | --- | --- | --- |
| Midwife characteristics | | | | |
| Age (mean, SD) | 47 | 8 | 46 | 9 |
| Gender Female (n, %) | 19 | 100.0% | 47 | 100.0% |
| Ethnic Group (n, %) |  |  |  |  |
| White | 19 | 100.0% | 45 | 95.7% |
| Other ethnic group | 0 | - | 1 | 2.1% |
| Prefer not to answer | 0 | - | 1 | 2.1% |
| Number of Years of Practice (mean, SD) | 21 | 16 | 19 | 10 |
| Currently employed (n, %) |  |  |  |  |
| Full time | 13 | 68.4% | 24 | 51.1% |
| Part Time | 6 | 31.6% | 23 | 48.9% |
| Area of practice (n, %) |  |  |  |  |
| Community midwife | 18 | 100.0% | 43 | 91.5% |
| Hospital-based midwife | 0 | - | 4 | 8.5% |
| Pre-intervention scores | | | | |
| Self-efficacy (mean, SD) |  |  |  |  |
| Communication-related behaviours | 68.8 | 15.2 | 71.1 | 16.4 |
| Support/intervention-related behaviours | 48.2 | 18.2 | 51.3 | 19.4 |
| Intention (mean, SD) |  |  |  |  |
| Communication-related behaviours | 86.9 | 15.5 | 86.3 | 13.9 |
| Support/intervention-related behaviours | 64.4 | 18.2 | 66.6 | 18.5 |
| Outcome expectancies (mean, SD) |  |  |  |  |
| Communication-related behaviours | 71.1 | 14.8 | 63.6 | 15.0 |
| Support/intervention-related behaviours | 67.8 | 13.5 | 70.4 | 17.8 |
| Behaviour (mean, SD) |  |  |  |  |
| Communication-related behaviours | 80.3 | 12.3 | 79.7 | 15.8 |
| Support/intervention-related behaviours | 50.9 | 15.7 | 52.8 | 19.7 |

*Note, 21 midwives only returned one questionnaire, two of these only returned their post-intervention questionnaire therefore have missing pre-intervention data.
