# Supplementary material for: Midwives’ survey of their weight management practice before and after the GLOWING guideline implementation intervention: A pilot cluster randomised controlled trial
Source: PLoS One. 2023 Jan 20;18(1):e0280624. doi: 10.1371/journal.pone.0280624 (PMC9858407; doi:10.1371/journal.pone.0280624)
Supplement: S5 Table — HW = healthy weight, OW = slightly overweight/very overweight *HW in the intervention arm includes one midwife who perceived their own weight as slightly underweight. (DOCX) [file pone.0280624.s007.docx]

**S6 Table: Midwives perceptions of the impact of their own weight on the level of difficulty the experience discussing weight status and risks of obesity with pregnant women**

|  | | Intervention (n, %) | | | Control (n, %) | | | Total sample (n, %) |
| --- | --- | --- | --- | --- | --- | --- | --- | --- |
|  |  | **HW*** | **OW** | **Total** | **HW** | **OW** | **Total** |  |
| Does your own weight make it easier or harder to discuss weight status with pregnant women with a BMI in the obese range? | Harder (1-3) | 5 (45.5) | 3 (15.0) | 8 (25.8) | 4 (25.0) | 4 (22.2) | 8 (23.5) | 16 (24.6) |
|  | No difference (4) | 5 (45.5) | 3 (15.0) | 8 (25.8) | 7 (43.8) | 6 (33.3) | 13 (38.2) | 21 (32.3) |
|  | Easier (5-7) | 1 (9.0) | 14 (70.0) | 15 (48.4) | 5 (31.3) | 8 (44.4) | 13 (38.2) | 28 (43.1) |
| Does your own weight make it easier or harder to discuss risks with pregnant women with a BMI in the obese range? | Harder (1-3) | 3 (27.3) | 1 (5.3) | 4 (13.3) | 1 (6.3) | 3 (16.7) | 4 (11.8) | 8 (12.5) |
|  | No difference (4) | 5 (45.5) | 4 (21.1) | 9 (30.0) | 8 (50.0) | 7 (38.9) | 15 (44.1) | 24 (37.5) |
|  | Easier (5-7) | 3 (27.3) | 14 (73.7) | 17 (56.7) | 7 (43.8) | 8 (44.4) | 15 (44.1) | 32 (50.0) |

HW = healthy weight, OW = slightly overweight/very overweight

*HW in the intervention arm includes one midwife who perceived their own weight as slightly underweight
