# Supplementary material for: Midwives’ survey of their weight management practice before and after the GLOWING guideline implementation intervention: A pilot cluster randomised controlled trial
Source: PLoS One. 2023 Jan 20;18(1):e0280624. doi: 10.1371/journal.pone.0280624 (PMC9858407; doi:10.1371/journal.pone.0280624)
Supplement: S6 Table — *Communication-related behaviours include weight communication and risk communication; support/intervention-related behaviours include diet and nutrition, physical activity, weight management, and referrals and signposting. (DOCX) [file pone.0280624.s008.docx]

**S7 Table. Internal validity of the questionnaire items for each behaviour category and social cognitive theory construct**

| **SCT Construct** | **Behaviour category*** | **Number of items** | **Cronbach’s Alpha** |
| --- | --- | --- | --- |
| Behaviour | Communication-related behaviours | 10 | 0.82 |
|  | Support/intervention-related behaviour | 22 | 0.94 |
| Self-efficacy | Communication-related behaviours | 7 | 0.95 |
|  | Support/intervention-related behaviour | 14 | 0.96 |
| Outcome expectancies | Communication-related behaviours | 10 | 0.90 |
|  | Support/intervention-related behaviour | 9 | 0.89 |
| Intentions | Communication-related behaviours | 10 | 0.91 |
|  | Support/intervention-related behaviour | 22 | 0.96 |

*Communication-related behaviours include weight communication and risk communication; support/intervention-related behaviours include diet and nutrition, physical activity, weight management, and referrals and signposting.
