# Supplementary material for: Midwives’ survey of their weight management practice before and after the GLOWING guideline implementation intervention: A pilot cluster randomised controlled trial
Source: PLoS One. 2023 Jan 20;18(1):e0280624. doi: 10.1371/journal.pone.0280624 (PMC9858407; doi:10.1371/journal.pone.0280624)
Supplement: S7 Table — SD = standard deviation * Due to the limited number of diet and nutrition questions for self-efficacy and outcome expectancies, these were combined with the physical activity questions when creating the sum scores for these constructs. (DOCX) [file pone.0280624.s009.docx]

**S8 Table: A comparison of pre- and post-intervention scores for communication- and support/intervention-related behaviour sub-categories and social cognitive theory constructs**

| Behaviour category | SCT construct | Behaviour sub-category | Intervention** | | Control** | | Pre-post intervention difference***  (mean difference, 95% CI) |
| --- | --- | --- | --- | --- | --- | --- | --- |
|  |  |  | **Pre-intervention (mean, SD)** | **Post-intervention (mean, SD)** | **Pre-intervention (mean, SD)** | **Post-intervention (mean, SD)** |  |
| Communication-related | Self-efficacy | Weight communication | 67.2 (21.1) | 76.3 (16.7) | 70.8 (18.2) | 71.1 (21.6) | 8.79 (-3.52, 21.10) |
|  |  | Risk communication | 68.6 (14.9) | 79.4 (16.4) | 74.1 (16.4) | 73.8 (18.8) | 9.05 (-0.89, 19.00) |
|  | Outcome expectancies | Weight communication | 69.8 (16.4) | 73.9 (18.9) | 64.6 (16.9) | 65.6 (21.1) | 4.46 (-5.79, 14.72) |
|  |  | Risk communication | 65.7 (14.3) | 72.4 (16.6) | 64.1 (16.6) | 62.7 (17.2) | 7.32 (-2.00, 16.64) |
|  | Intention | Weight communication | 90.1 (15.2) | 92.9 (8.7) | 89.1 (14.1) | 90.5 (12.6) | 2.06 (-5.10, 9.22) |
|  |  | Risk communication | 85.1 (14.2) | 89.3 (15.7) | 81.9 (17.0) | 82.2 (16.3) | 4.05 (-3.86, 11.96) |
|  | Behaviour | Weight communication | 88.1 (13.2) | 85.8 (13.7) | 86.5 (18.3) | 85.5 (12.9) | -3.13 (-11.47, 5.21) |
|  |  | Risk communication | 73.8 (16.1) | 83.6 (14.3) | 71.2 (19.6) | 71.5 (18.4) | 12.30 (3.07, 21.53) |
| Support and intervention-related | Self-efficacy | Diet, nutrition, physical activity* | 49.3 (16.5) | 76.4 (16.0) | 56.9 (19.7) | 58.6 (22.9) | 24.77 (14.09, 35.44) |
|  |  | Weight management | 48.3 (19.8) | 72.1 (18.3) | 52.5 (23.5) | 59.7 (19.3) | 18.88 (7.88, 29.88) |
|  |  | Referrals and signposting | 47.9 (17.3) | 63.3 (26.0) | 45.3 (24.6) | 55.1 (24.6) | 6.57 (-8.26, 21.39) |
|  | Outcome expectancies | Diet, nutrition, physical activity* | 66.1 (23.8) | 78.3 (18.6) | 74.0 (20.2) | 73.8 (17.9) | 8.68 (-5.99, 23.35) |
|  |  | Weight management | 71.0 (22.7) | 79.3 (18.3) | 77.0 (17.7) | 73.8 (16.4) | 8.68 (-4.03, 21.39) |
|  |  | Referrals and signposting | 59.9 (26.8) | 70.0 (23.3) | 56.1 (19.7) | 55.4 (23.6) | 13.94 (-1.74, 29.63) |
|  | Intention | Diet and nutrition | 71.9 (19.5) | 88.1 (14.1) | 70.7 (20.0) | 77.7 (18.1) | 11.12 (0.99, 21.25) |
|  |  | Physical activity | 63.2 (19.8) | 88.1 (13.3) | 66.6 (21.4) | 72.2 (21.7) | 16.87 (5.72, 28.02) |
|  |  | Weight management | 62.1 (22.3) | 83.6 (14.0) | 60.7 (22.2) | 71.4 (20.5) | 10.30 (-1.65, 22.26) |
|  |  | Referrals and signposting | 70.7 (21.6) | 78.9 (28.4) | 73.9 (20.1) | 76.6 (19.9) | 9.13 (-9.91, 28.16) |
|  | Behaviour | Diet and nutrition | 66.0 (20.0) | 71.5 (13.2) | 67.3 (19.2) | 70.8 (20.8) | 2.42 (-10.06, 14.89) |
|  |  | Physical activity | 44.9 (17.0) | 61.6 (21.2) | 51.1 (24.4) | 59.0 (24.2) | 7.47 (-4.23, 19.18) |
|  |  | Weight management | 43.0 (19.4) | 58.9 (20.3) | 46.1 (27.0) | 54.5 (21.2) | 7.36 (-5.50, 20.22) |
|  |  | Referrals and signposting | 56.4 (18.4) | 57.4 (25.3) | 67.6 (23.1) | 65.4 (20.0) | 4.29 (-9.64, 18.23) |

SD = standard deviation, CI = confidence interval

* Due to the limited number of diet and nutrition questions for self-efficacy and outcome expectancies, these were combined with the physical activity questions when creating the sum scores for these constructs

** Mean and SD calculated for all midwives returning any questionnaire

*** Mean difference and 95% CI calculated for midwives who returned both pre- and post-intervention questionnaires
