# Supplementary material for: Midwives’ survey of their weight management practice before and after the GLOWING guideline implementation intervention: A pilot cluster randomised controlled trial
Source: PLoS One. 2023 Jan 20;18(1):e0280624. doi: 10.1371/journal.pone.0280624 (PMC9858407; doi:10.1371/journal.pone.0280624)
Supplement: Attachment — Submitted filename: renamed_72c09.docx [file pone.0280624.s010.docx]

**COMMENTS – Manuscript PONE-D-21-39444**

Title: “Midwives’ survey of their weight management practice before and after the GLOWING guideline implementation intervention: a pilot cluster randomised controlled trial”

**Important note: This review pertains only to ‘statistical aspects’ of the study and so** **‘clinical aspects’ [like medical importance,** **relevance of the study,** **‘clinical significance and implication(s)’ of the whole study, etc.] are to be evaluated [should be assessed] separately/independently. Further please note that any ‘statistical review’ is generally done under the assumption that (such) study specific methodological [as well as execution] issues are perfectly taken care of by the investigator(s). This review is not an exception to that and so does not cover clinical aspects {however, seldom comments are made only if those issues are intimately / scientifically related & intermingle with ‘statistical aspects’ of the study}. Agreed that ‘statistical methods’ are used as just tools here, however, they are vital part of methodology [and so should be given due importance].**

**COMMENTS:** What exactly you want to convey when you say [in ‘Abstract-Background’] that “The GLOWING intervention uses social cognitive theory (SCT) to address evidence-based barriers to practice, aiming to support midwives’ implementation of guidelines” and later [in ‘Abstract-Methods’] say “UK guidelines were grouped into communication-related behaviours (weight- and risk-communication) and support/intervention-related behaviours (diet/nutrition, physical activity, weight management, referrals/signposting)” and also say that “randomised to intervention (all eligible midwives received the intervention) or control (no intervention)”. Is not this account confusing? At least for me it is confusing. I could not really be clear about why “UK guidelines were grouped into two” and which is the intervention? Please make me [average reader] understand clearly.

In fact, when the allocation is ‘randomly’ done, statistical comparison of baseline characteristics is not indicated. In this context, please read the following which is pasted from one standard textbook on ‘Research Methodology’.

Statistical comparison of baseline characteristics is not desirable at all [because even if P-value turns out to be significant (while comparing baseline characteristics despite random allocation), it is, by definition, a false positive] as you then are supposed to be testing ‘randomization’ then, which in any single trial may not balance all baseline characteristics because ‘randomization’ is a sort of ‘insurance’ and not a guarantee scheme.

Though the measures/tools used are appropriate [line 237-: SCT questionnaires], most of them yield data that are in [at the most] ‘ordinal’ level of measurement [and not in ratio level of measurement for sure {as the score two times higher does not indicate presence of that parameter/phenomenon as double (for example, a Visual Analogue Scales VAS score or say ‘depression’ score)}]. Then application of suitable non-parametric test(s) is/are indicated/advisable [even if distribution may be ‘Gaussian’ (i.e. normal)]. Agreed that there is/are no non-parametric test(s)/technique(s) available to be used as alternative in all situation(s) [suitable / most desired/applicable], but should be used whenever/wherever they are available.

This being a ‘pilot’ study/trial, ‘sample size’ is not an issue. However, from the account given in lines 43-44 [post-intervention, mean (SD) scores were consistently higher among intervention midwives than controls] it seems that you have compared post-intervention (for both) only. Is that by ANCOVA so that Pre-intervention/baseline scores are taken cognizance of? Even if baselines are similar {not significantly different}, one should take pre scores (baseline) into consideration. Why not work on ‘change score(s)’? According to section ‘Comparing intervention and control arms’ [lines 344-354: The descriptive statistics for pre- and post-intervention data are shown in Table 3, split by the communication- and support/intervention-related categories. The mean scores for the communication-related category were higher overall than for the support/intervention-related category. Communication-related behaviours and intentions were high in both the intervention and control arms, pre- and post-intervention, suggesting there may be a ceiling effect for these constructs. In the control arm, there was limited difference between the pre- and post-intervention scores for both behaviour categories. Post-intervention, the scores were consistently higher in the intervention arm than the control arm, particularly for support/intervention self-efficacy (mean scores 71.4 (SD 17.1) and 58.4 (SD 20.1) respectively) and intentions (mean scores 85.4 (SD 13.7) and 73.6 (SD 18.7) respectively)] you have compared ‘pre- and post-intervention scores’ separately for both groups. Whereas the section heading ‘Comparing intervention and control arms’ implies/indicates between groups comparison.

May please read the following which is again pasted from the same textbook on ‘Research Methodology’.

Many randomized trials involve measuring at baseline and after treatment. In few papers [example: *‘The use of percentage change from baseline as an outcome in a controlled trial is statistically inefficient – a simulation study’ by Andrew Vickers in BMC Medical Research Methodology 2001, 1:6*], compared all four possibilities for how such trials can be analyzed: only post–treatment; absolute change between baseline and post-treatment (called as ‘change score’ method); percentage change between baseline and post-treatment (called as ‘percent change score’ method) and analysis of covariance (ANCOVA) with baseline score as a covariate. The statistical power of each method was determined for a hypothetical randomized trial under a range of correlations between baseline and post-treatment scores. ANCOVA has been found to have the highest statistical power.

‘Strengths and limitations’ of this research given in lines 459 onwards are appreciated. Though, as pointed out in ‘important note’ above “This review pertains only to ‘statistical aspects’ of the study and so ‘clinical aspects’ should be assessed separately/independently, in my opinion, this study/article has great(er) potential and to rescue this article (which is quite possible), some amount of re-vision/re-drafting (may be considering above highlighted points) is needed, I guess.
